# Crossmodal Associations with Olfactory, Auditory, and Tactile Stimuli in Children and Adults

**DOI:** 10.1177/20416695211048513

**Published:** 2021-12-06

**Authors:** Laura J. Speed, Ilja Croijmans, Sarah Dolscheid, Asifa Majid

**Affiliations:** Centre for Language Studies, Radboud University, Nijmegen, The Netherlands; Faculty of Social and Behavioral Sciences, Utrecht University, Utrecht, The Netherlands; Department of Rehabilitation and Special Education, 14309 University of Cologne, Cologne, Germany; Biopsychology & Cognitive Neuroscience, Faculty of Psychology & Sports Science, 9167 Bielefeld University, Bielefeld, Germany; Department of Psychology, University of York, York, UK

**Keywords:** crossmodal associations, crossmodal correspondence, multisensory development, musical pitch, touch, odor

## Abstract

People associate information with different senses but the mechanism by which this happens is unclear. Such associations are thought to arise from innate structural associations in the brain, statistical associations in the environment, via shared affective content, or through language. A developmental perspective on crossmodal associations can help determine which explanations are more likely for specific associations. Certain associations with pitch (e.g., pitch–height) have been observed early in infancy, but others may only occur late into childhood (e.g., pitch–size). In contrast, tactile–chroma associations have been observed in children, but not adults. One modality that has received little attention developmentally is olfaction. In the present investigation, we explored crossmodal associations from sound, tactile stimuli, and odor to a range of stimuli by testing a broad range of participants. Across the three modalities, we found little evidence for crossmodal associations in young children. This suggests an account based on innate structures is unlikely. Instead, the number and strength of associations increased over the lifespan. This suggests that experience plays a crucial role in crossmodal associations from sound, touch, and smell to other senses.

## Introduction

Objects and events are processed in multiple perceptual modalities simultaneously. Corresponding perceptual features across modalities are bound together to form object and event representations (Evans & Treisman, 2011), and are therefore associated. Crossmodal associations (i.e., associations between perceptual modalities) have been demonstrated between vision and taste (e.g., between the color red and sweetness; O'Mahony, 1983), the texture of sandpaper and spiky-sounding words (Etzi et al., 2016), visual lightness and high vibrotactile frequency (Martino & Marks, 2000), and temperature and color (Spence, 2020b), to give but a few examples.

Although fascinating in their own right, it is unclear how these crossmodal associations emerge and change over development. At least four possible mechanisms have been proposed (e.g., Spence, 2011), which are not necessarily mutually exclusive. First, correspondences between modalities may be the result of innate connections in the brain (i.e., structural associations). Such associations should therefore be present at birth. This resonates with the neonatal synaesthesia hypothesis (Maurer & Maurer, 1988; for review see Deroy & Spence, 2013a), which proposes that all newborns possess synaesthetic associations between the senses. Second, crossmodal associations may be scaffolded by environmental co-occurrences (i.e., statistical associations). For example, there are environmental associations between sound and space which could be extracted from experience: high pitch sounds typically occur higher in space (Parise et al., 2014), and the human larynx rises when producing high-frequency sounds (Parkinson et al., 2012). Accordingly, statistical associations should emerge throughout development as experience accumulates. The third mechanism is via language: language may play a role in the development and reinforcement of such associations (i.e., semantic associations in language). For example, in some languages pitch is described with the spatial terms *thin* and *thick*, and these labels could be used to promote crossmodal mappings (Dolscheid et al., 2013; Shayan et al., 2014). Associations based on semantics should develop after language acquisition and may differ across languages. A final account suggests that crossmodal associations are mediated by emotion (Deroy et al., 2013; Palmer et al., 2013; Spence, 2020a). According to this account, associations may be based on shared affective content.

Two types of associations that appear to have different developmental trajectories for crossmodal associations are auditory pitch and tactile properties. There is evidence that crossmodal associations with pitch become stronger with age (Cuturi et al., 2019; Starr & Srinivasan, 2018). This could occur if associations are learned from statistical regularities in the environment (such as pitch and height; Parise et al., 2014), or if they are strengthened by language (e.g., by describing pitch with *thin* and *thick*; Shayan et al., 2014; see also Dolscheid et al., 2020; Nava et al., 2016; Starr et al., 2020; Starr & Srinivasan, 2018). On the other hand, it has been suggested that associations between tactile stimuli and chroma decline with age: Ludwig and Simner (2013) found significant associations between chroma and tactile properties in children but not adults, suggesting innateness of associations (cf., neonatal synaesthesia^1^; Maurer & Mondloch, 2005), which is lost during development through processes such as neural pruning.

One modality for which the developmental trajectory of crossmodal associations has not been as well studied is olfaction. Although previous studies have investigated odor associations (e.g., Belkin et al., 1997; Crisinel et al., 2013; Spence, 2020c; Deroy et al., 2013; de Valk et al., 2017) and the development of odor identification (Cain et al., 1995; Cavazzana et al., 2017; Goubet et al., 2014; Lehrner et al., 1999; Monnery-Patris et al., 2009), few studies have systematically investigated associations between odors and a range of perceptual modalities using real perceptual stimuli in the same participants. Previous studies have focused on associations between odors and a single dimension (e.g., color; see Goubet et al., 2018), or have used words instead of perceptual stimuli (e.g., texture words; Spector & Maurer, 2012). It is surprising that odor has been relatively neglected, since crossmodal associations with an odor should be particularly strong due to the salience of color and shape in food and drinks, as well as the packaging and branding of products. However, compared to the well-documented crossmodal associations in the domains of vision and audition, it has been said that odor crossmodal associations (particularly with sounds and shapes) may be different. They may be less relevant in multisensory interactions, less likely to be caused by statistical associations, and less likely to be driven by linguistic factors (Deroy et al., 2013).

Odor crossmodal associations may be different from crossmodal associations in vision and audition in part due to differences between odor cognition versus visual and auditory cognition. For example, odor naming is particularly challenging for most people (Cain, 1979; Majid et al., 2018), and relies heavily on source-based naming (e.g., *smells like banana*), although some communities do use abstract terms for odor naming (e.g., Majid & Burenhult, 2014; Majid & Kruspe, 2018). Furthermore, olfactory cognition shows a different profile to the other senses in both episodic (e.g., Engen & Ross, 1973) and autobiographical odor memory (Willander & Larsson, 2006), as well as olfactory imagery (Arshamian et al., 2020; Arshamian & Larsson, 2014), suggesting odors are thought about in a fundamentally different way to stimuli from the dominant modalities. For example, while imagery for vision and audition becomes stronger over development, olfactory imagery remains weak in adulthood (Arshamian et al., 2020). This implies that patterns of crossmodal associations to odor could also be qualitatively different.

The current paper, therefore, examines crossmodal associations with odors across development and across a range of perceptual dimensions. In addition, we explore the pattern of crossmodal associations with pitch and tactile properties, two modalities that previous research indicates should have different developmental trajectories. By doing so, we attempt to compare the role of different sensory modalities in the development of crossmodal associations and therefore build on a growing body of work that emphasizes the importance of comparing across perceptual modalities in a concerted fashion (e.g., Levinson & Majid, 2014; Majid et al., 2018).

Widening the scope of existing studies then, we conducted three experiments assessing crossmodal associations with auditory pitch, tactile stimuli, and odors, and a range of perceptual dimensions that have so far been studied separately. For pitch, we assessed dimensions previously shown to have consistent crossmodal associations with pitch—shape (Marks et al., 1987), brightness (Marks, 1974; Marks et al., 1987), spatial height (Bernstein & Edelstein, 1971; Dolscheid et al., 2014; Dolscheid et al., 2020; Nava et al., 2016; Starr & Srinivasan, 2018), size (Gallace & Spence, 2006; Marks, 1974; Parise & Spence, 2012), and thickness (Dolscheid et al., 2020; Shayan et al., 2014; Starr & Srinivasan, 2018). We also tested crossmodal associations between haptic sharpness and weight with pitch. These have previously been assessed primarily with linguistic materials (Eitan & Rothschild, 2011; Eitan & Timmers, 2010; Walker & Smith, 1985), and only recently with real perceptual stimuli (Hamilton-Fletcher et al., 2018; Walker et al., 2017).

In the tactile task, we included visual dimensions previously shown to be associated with tactile properties: hue, chroma, and lightness (Jraissati et al., 2016; Ludwig & Simner, 2013; Martino & Marks, 2000; Slobodenyuk et al., 2015). We also tested associations with shape and pitch. It has been shown that after mouthing a hard or soft pacifier, one-year-olds prefer to look at a pacifier with a shape depicting a novel texture (Gibson & Walker, 1984), suggesting children may be sensitive to tactile–shape associations early. It has also been shown that adults make consistent associations between oral texture and shape, and oral texture and pitch (Spence & Gallace, 2011), and people associate pitch with tactile objects and tactile-related words (Walker & Smith, 1984, 1985; p. 1986).

For the odor task, we tested crossmodal associations with dimensions that have previously been investigated—pitch (Belkin et al., 1997; Crisinel & Spence, 2012a), shape (Crisinel et al., 2013; Hanson-Vaux et al., 2013; Seo et al., 2010; Smets & Overbeeke, 1989), and hue (de Valk et al., 2017; Gilbert et al., 1996; Goubet et al., 2018; Kim, 2013; Levitan et al., 2014; Maric & Jacquot, 2013; Speed & Majid, 2018). We also included tactile stimuli, for which explicit crossmodal associations to odor have only been assessed with words (Spector & Maurer, 2012; although there is evidence that odors affect perceived texture, supporting the existence of odor–tactile associations; e.g., Croy et al., 2014; Demattè et al., 2006; Krishna et al., 2010).

We explored these associations in a large group of participants across a wide age range. In contrast to many studies, we focus on development across the lifespan from age four years into adulthood, rather than testing only young children, as is often the case (see, e.g., Dolscheid et al., 2014; Fernandez & Bahrick, 1994; Haryu & Kajikawa, 2012; Mondloch & Maurer, 2004; Nava et al., 2016; Shayan et al., 2014; Starr & Srinivasan, 2018; Wada et al., 2012; Walker et al., 2010). Development does not end in early childhood, and by exploring associations in older children and adults, we can assess changes that occur with accruing experience.

Based on previous studies, we propose several hypotheses. Associations of pitch with spatial height, thickness, size, and brightness, should be observed from an early age (Dolscheid et al., 2014; Mondloch & Maurer, 2004; Shayan et al., 2014; Walker et al., 2010), but also increase with age (e.g., Cuturi et al., 2019; Dolscheid et al., 2020; Starr & Srinivasan, 2018). In contrast, associations between tactile dimensions and chroma should be observed early but reduce with age since they are thought to be innate associations that diminish over time due to neural pruning (Ludwig & Simner, 2013). For other associations, the investigation is more exploratory. Whether or not the developmental trajectory of associations between tactile properties and other dimensions (i.e., shape and pitch) is similar to those with chroma is unclear. It is also difficult to make predictions for the developmental trajectory of odor associations since some aspects of olfactory perception appear innate and stable over time (Khan et al., 2007; Soussignan et al., 1997; although see, e.g., Logue et al., 1988; Maier-Nöth et al., 2016), and for others, the strong influence of experience on olfaction has also been emphasized (Brennan & Keverne, 1997; Goubet et al., 2018, 2014; Shankar et al., 2010; Wilson & Stevenson, 2006). If crossmodal associations are driven by experience, such as statistical associations or semantic information, then associations should be more robust in older participants (as suggested previously by Goubet et al., 2014). On the other hand, if crossmodal associations are less susceptible to accruing experience, associations should be similar across age groups.

All experiments were conducted in Nemo Science Museum in Amsterdam, the Netherlands. Our study, therefore, can also be seen as a test of whether crossmodal associations observed in previous studies generalize from the controlled laboratory setting to noisier contexts and crucially with a more diverse participant sample (see Hruschka et al., 2018; Speed et al., 2018). All participants had the opportunity to take part in all three experiments but not all did, so the samples of participants for each experiment were not identical. In addition, the current experiments were designed based on previous studies targeting specific modalities, therefore experimental design and procedures differ somewhat across the three experiments. We therefore present the methods and results for each experiment separately, beginning with (Section “Experiment 1: Auditory Pitch”) auditory pitch, (Section “Experiment 2: Tactile Mappings”) tactile mappings, and finally (Section “Experiment 3: Odor”) odor. The experimental protocols were approved by the local Ethics Assessment Committee of the Centre for Language Studies at Radboud University, and by Science Museum Nemo, Amsterdam.

## Experiment 1: Auditory Pitch

### Method

#### Participants

Four-hundred and sixty-six participants volunteered to take part (251 females, 3 participants’ gender was not recorded, age *M*  =  25.99 years, *SD*  =  18.38, range  =  4–81 years). Participants represented native speakers of 24 different languages, with the most common languages being Dutch (66%), English (9%), German (8%), and French (4%). All participants were visitors to Nemo Science Museum. Informed consent was obtained from participants.

#### Material

For auditory stimuli, two pairs of sounds consisting of a high and low pitch tone were used (330 Hz vs. 262 Hz and 659 Hz vs. 440 Hz), with loudness matched across tones at ∼60 dB-a. We assessed crossmodal associations from pitch to dimensions in visual and tactile modalities. We used simple shapes to investigate the following visual dimensions: thickness (thick–thin), size (big–small), height (high space–low space), brightness (bright–dull), and angularity (spiky–rounded). Images of eyes were added to all shapes (except for angularity) to make the task more engaging for children. In the tactile modality, we investigated the dimensions of sharpness (sharp–blunt) and weight (heavy–light). For sharpness judgments, two polystyrene objects were used: one cone-shaped for “sharp,” and one egg-shaped for “blunt.” For weight, two small plastic containers were used: one was filled with modeling clay to weigh more than the other (70 g vs. 10 g). Objects for the tactile judgments were placed inside opaque boxes, with a small opening to fit a hand inside. Participants could not see the objects. Dimensions were counterbalanced across participants (left vs. right), except for sharpness and weight judgments because boxes needed to be in a fixed position.

#### Procedure

The experiment took place in a small room inside Nemo Science Museum, as part of a “Science Live” event. A maximum of two other participants were tested at the same time at different testing stations. Data were collected with a laptop running E-Prime. Participants could complete a Dutch, English, or German version of the experiment. The instructions remained the same, regardless of language (comparability was ensured through back-translation).

**Figure 1.**
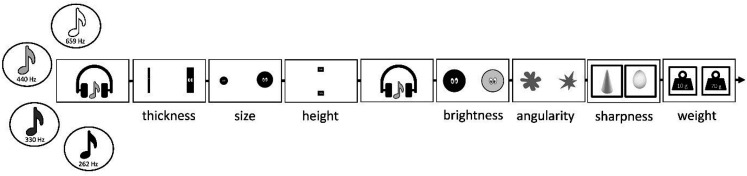
Schematic visualization of Experiment 1. For each tone, participants chose which stimulus they associated it with for each dimension in the following order: thickness, size, height, brightness, angularity, sharpness, and weight. For sharpness and weight, objects were placed inside boxes.

At the start of the experiment, participants heard one high pitch sound and one low pitch sound, so they were familiar with the auditory dimension of interest. On individual trials, each sound was played for 2 s, and then participants had to choose which stimulus they associated with the sound for each dimension in the following order: thickness, size, height, brightness, angularity, sharpness, and weight (see Figure 1). For each judgment, options appeared on the left and right of the screen (e.g., the spiky shape on the left and the rounded shape on the right), with the side of the screen counterbalanced. For judgments of sharpness and weight, participants placed their hands inside the boxes and felt the objects. Response options stayed on the screen until the participant clicked on their choice with the mouse. The image selected was then highlighted in red for 1 s. After three judgments, participants listened to the sound again for 2 s. During the course of the experiment, participants heard each of the four sounds and completed judgments on all dimensions per sound before moving on to the next one. The order of sounds was randomized and therefore sounds were not presented as pairs. The whole task took around 5 min.

### Results

Eight participants were removed from the study based on notes taken by research assistants during testing because they did not follow instructions properly, experienced computer problems, and one participant was autistic (as stated by the parent). Ten participants were removed because they did not provide demographic information. The remaining participants were divided into four age groups: 4–5 years (*n*  =  12), 6–9 years (*n*  =  87), 10–18 years (*n*  =  116), and 19 years and older (*n*  =  233). This division roughly corresponds to that of Ludwig & Simner (2013), except we included an additional 4- to 5-year-old group since our sample contained younger children.

For each dimension (e.g., spiky-rounded) the number of counts for one choice (e.g., spiky) was calculated separately for each pitch. This meant there were separate scores for high pitch and low pitch sounds (min  =  0, max  =  2). We therefore analyzed choices for each pitch and dimension separately using one sample Wilcoxon Signed Rank tests which are appropriate for ordinal data to test whether counts were significantly different from the median of the possible counts (i.e., 1). We analyzed the data this way because we do not wish to assume a priori that choices are bipolar. For example, if participants significantly match high pitch sounds to spiky shapes, it does not necessarily entail they would also match low pitch sounds to rounded shapes more than chance. We first analyzed the data collapsed across age groups. The alpha level was adjusted with Bonferroni correction, based on the overall number of tests conducted (i.e., 0.05/14  =  0.004)

We found high pitch sounds were significantly matched to thin, small, bright, spiky, sharp, and light objects, whereas low pitch sounds were significantly matched to thick, big, rounded, blunt, and heavy objects (see Table 1). Neither high nor low pitch sounds were matched to high and low space more than chance, although the mappings were in the predicted direction.

**Table 1.** Wilcoxon Signed-Rank Tests for High and Low Pitch on Each Dimension.

	High	Low
Thickness	*z* = 11.36, *p* < .001*	*z* = 8.01, *p* < .001*
Size	*z* = 10.57, *p* < .001*	*z* = 5.77, *p* < .001*
Height	*z* = 7.37 *p* < .001*	*z* = .77, *p* = .44
Brightness	*z* = 11.41, *p* < .001*	*z* = 2.69, *p* = .007
Angularity	*z* = 5.97, *p* < .001*	*z* = 10.42, *p* < .001*
Sharpness	*z* = 8.67, *p* < .001*	*z* = 8.03, *p* < .001*
Weight	*z* = 8.32, *p* < .001*	*z* = 5.85, *p* < .001*

*Note*. Asterisks Denote p < .004 (Bonferroni-corrected Alpha).

**Figure 2.**
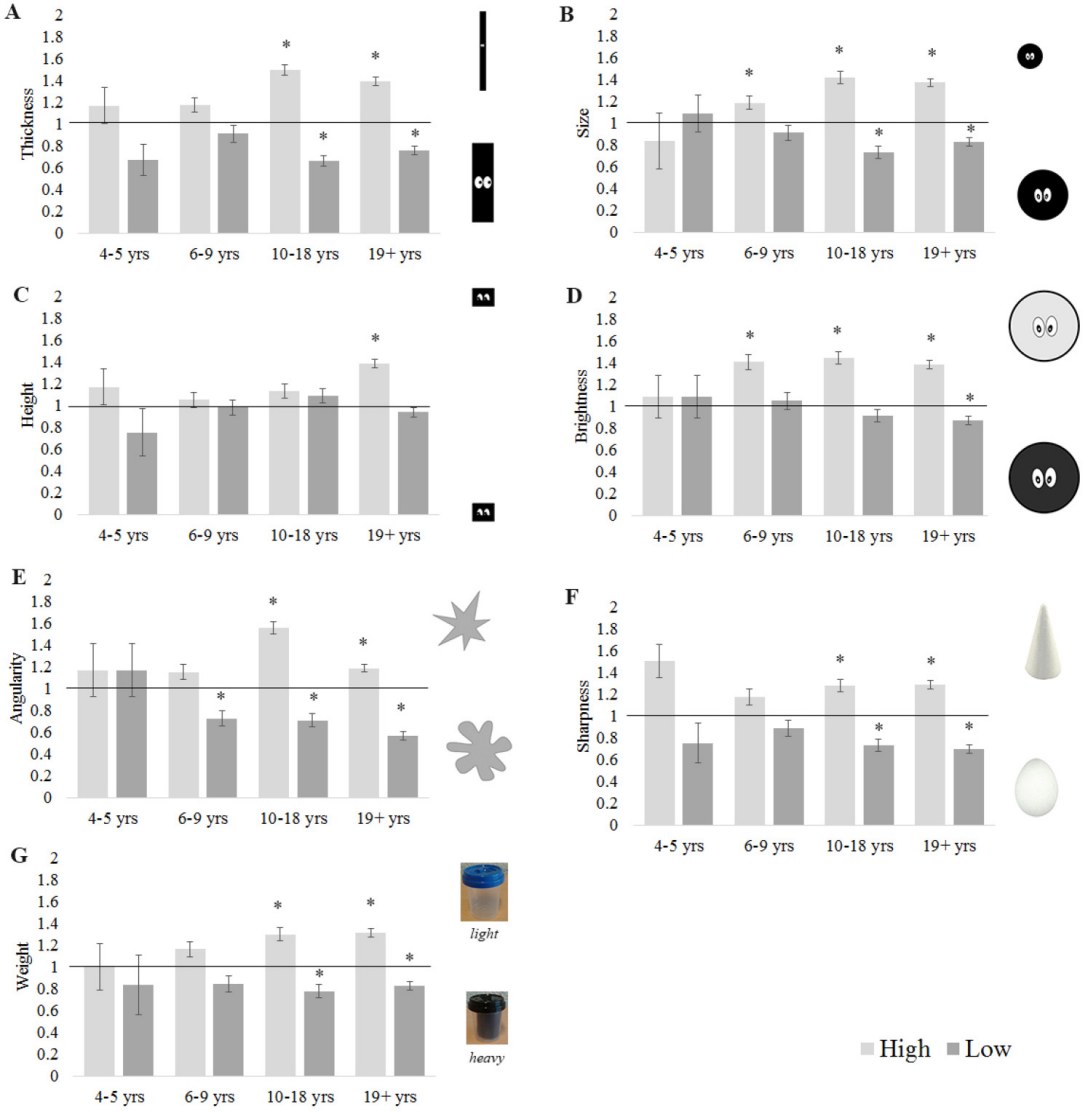
For each age group, mean count across participants of matches to (A) thin, (B) small, (C) high, (D) bright, (E) spiky, (F) sharp, and (G) lightweight objects. Asterisk indicates matches greater than chance (Bonferroni-corrected alpha *p* < .004). Error bars equal to 1 *SE*.

We then tested whether counts for high and low pitch differed significantly from chance separately for each age group (statistics are reported in Table A1 in Appendix A; see Figure 2). Overall, our results show that crossmodal associations with pitch become stronger with age. All associations (except for height-pitch) resulted in above chance performance for the cohort of 19 + years, but differed in their onset for the younger age groups.

Although we did not want to assume mappings were bipolar, it is still informative to know whether a difference exists between high and low pitch for each dimension. We therefore tested whether associations differed between high and low pitch across age groups. We conducted Wilcoxon signed-rank tests to compare mappings between high and low pitch for each dimension and each age group. The alpha level was adjusted with Bonferroni correction based on the overall number of tests conducted per dimension (i.e., 0.05/4  =  0.0125) (See Table A2 in Appendix A for corresponding statistics. We also analyzed data with age as a continuous variable which can be found in the Supplemental Materials). For the following analyses, we compared high to low pitch sounds for their likelihood of being matched to various dimensions.

Considering the various visuospatial dimensions, beginning with thickness, there was a significant difference between high and low pitch at all ages, but using the adjusted alpha value the difference was not significant in the two youngest age groups (i.e., in children up to 9 years old). For the other two age groups, high pitch sounds were more likely than low pitch sounds to be matched to the thin object. There was no difference between high and low pitch for the youngest age group (age 4–5 years) for mapping with size, but in the other three age groups high pitched sounds were matched significantly more to the small object. For height mappings, the only significant difference between high and low pitch was found for the oldest group (19 years and older), where the high-pitch tone was matched significantly more to high space.

Moving on to brightness, there was no difference between high and low pitch for the youngest age group (age 4–5 years) for brightness mappings, but in the other three age groups high pitched sounds were matched significantly more to the light object. Similarly, for angularity, there was no difference between high and low pitch for the youngest age group (age 4–5 years), but in the other three age groups high pitched sounds were matched significantly more to the spiky object.

Turning to the tactile dimensions, for sharpness, there was a significant difference between high and low pitch at all ages, but using the adjusted alpha value the difference was not significant in the two youngest age groups (i.e., in children up to 9years-old). For the other two age groups, high pitch sounds were more likely to be matched to the sharp object. Finally, for weight, there was no difference between high and low pitch for the youngest age group (age 4–5 years), but in the other three age groups high pitched sounds were matched significantly more to the light object than low pitch sounds were.

### Summary

Overall, high pitch sounds were matched to objects that were thin, small, bright, spiky, sharp, and light; whereas low pitch sounds were matched to objects that were thick, big, rounded, blunt, and heavy. These associations are in line with those observed earlier (e.g., Gallace & Spence, 2006; Karwoski et al., 1942; Marks, 1974; Marks et al., 1987). In addition, this is the first study to behaviorally demonstrate associations between pitch and tactile sharpness, which have previously only been assessed with linguistic materials, i.e., adjectives (Eitan & Rothschild, 2011; Eitan & Timmers, 2010), but not perceptual stimuli.

Neither high pitch nor low pitch was significantly matched to high or low space, except in the adult group, and even then only high pitch showed a reliable association with high space. This is puzzling at first considering the number of existing demonstrations of a pitch–height mapping (e.g., Dolscheid et al., 2014; Starr & Srinivasan, 2018), however other studies suggest this mapping may be more fragile than previously realized (see, e.g., Dolscheid et al., 2020; Nava et al., 2016). This discrepancy could be the result of the specific stimuli used. It has been suggested that pitch–height associations occur for only part of the pitch range (Parise et al., 2014), for example. There are also differences in how spatial stimuli are realized. For example, in our experiment, we placed eyes on the objects to make them more engaging for children. Although the stimuli do not depict a specific emotion—which is known to affect associations (Woods et al., 2013)—the eyes did look in a leftwards direction, which may have drawn attention away from the vertical dimension. This is something that could be systematically explored in future studies.

In general, more associations with pitch were observed as people aged, suggesting these associations are strengthened through development. Our results therefore align with previous findings showing that crossmodal pitch associations become stronger with age (e.g., Starr & Srinivasan, 2018). In the same vein, there is evidence to suggest that crossmodal associations become more fine-grained across development (e.g., Cuturi et al., 2019). For instance, Cuturi et al. (2019) tested 6- to 9-year-old children in crossmodal association tasks where they had to match tones of different pitches to circles that best matched the tones in size. Whereas younger children preferably opted for extreme sizes, older children chose more intermediate sizes, demonstrating crossmodal associations become more fine-tuned with age. Although our study did not manipulate continuous spatial (and other) stimulus dimensions, the comparatively small pitch range we used could be interpreted in a similar way: while very young children and even infants seem to be able to make crossmodal pitch associations when pitch differences are large (e.g., pitch ranges between 300 and 1700 Hz in Dolscheid et al., 2014 and Walker et al., 2010), only older children and adults seem to reliably form crossmodal associations when smaller pitch ranges are used (as in our study). Although suggestive, clearly more evidence is needed to test this proposal.

In sum, although null effects in the youngest age group could reflect a lack of power, our findings add further evidence to recent proposals that crossmodal pitch associations are not fixed, but become more consistent with age.

## Experiment 2: Tactile Mappings

### Method

#### Participants

Four-hundred and sixty-four participants volunteered to take part in the experiment (242 females, 3 participants’ gender was not recorded, age *M*  =  25.95 years, *SD*  =  18.27, range =  4–81 years). All other details are the same as Experiment 1.

#### Materials

**Figure 3.**
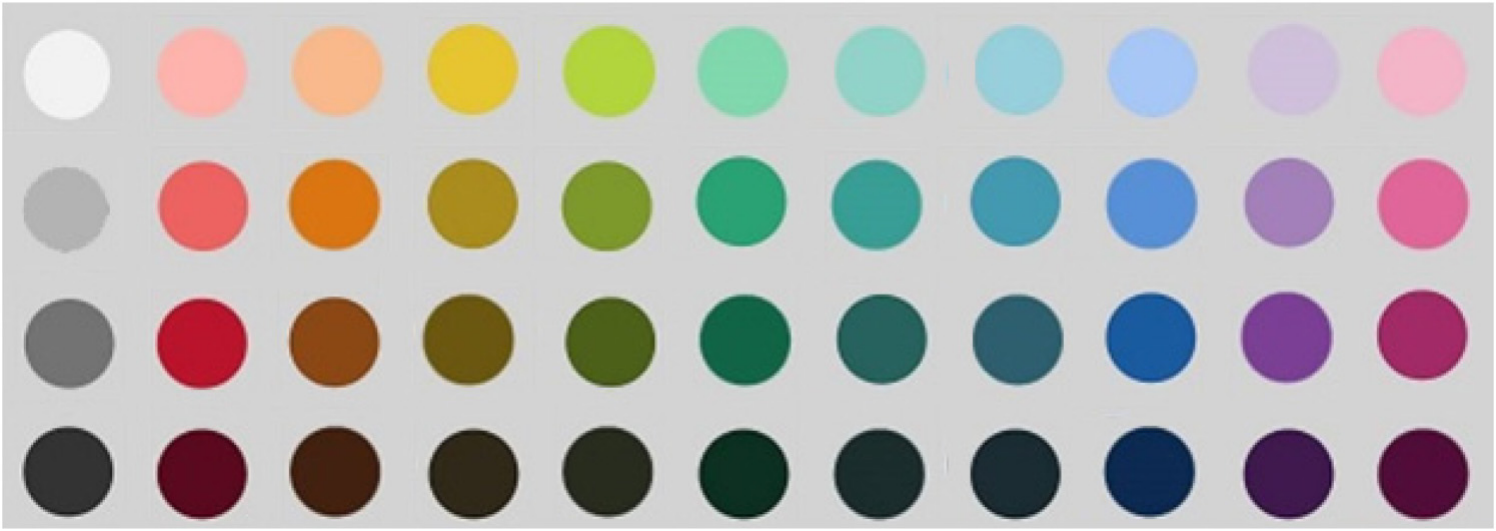
Approximation of colors used in experiments 2 and 3.

**Figure 4.**
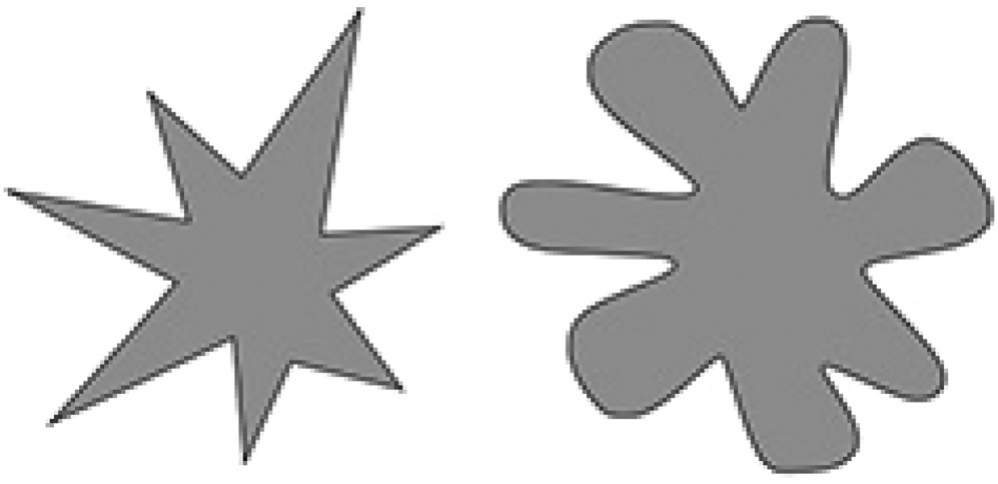
Spiky and rounded shapes used in experiments 2 and 3.

We used three tactile scales, following Ludwig and Simner (2013). However, to keep the duration of the task short, two rather than six objects were used for each scale. A piece of sandpaper was used for “rough” and plastic for “smooth.” A poster tube wrapped in a pair of tights was “hard,” while a tube of tights filled with fabric was “soft.” Similar shapes and surface covering were used so that the two objects only differed in the crucial dimension of hardness–softness. Two polystyrene objects, one with a pointed top and one with a round top, were used as “sharp” and “blunt.” Each object was placed inside an opaque box containing a hole on one side in which a hand could enter. Forty-four Munsell colors were selected (following Majid & Levinson, 2007) to be presented on the screen (see Figure 3). Laptop monitors were color-calibrated using Spyder 5 screen calibration before the onset of testing every day. For mappings with pitch and shape, we used scales anchoring two opposites, following previous studies (e.g., Spence & Gallace, 2011). For mappings with pitch, one high pitch (330 Hz) and one low pitch (262 Hz) tone were used. For shape mappings, a greyscale spiky and rounded shape were used (see Figure 4).

#### Procedure

**Figure 5.**
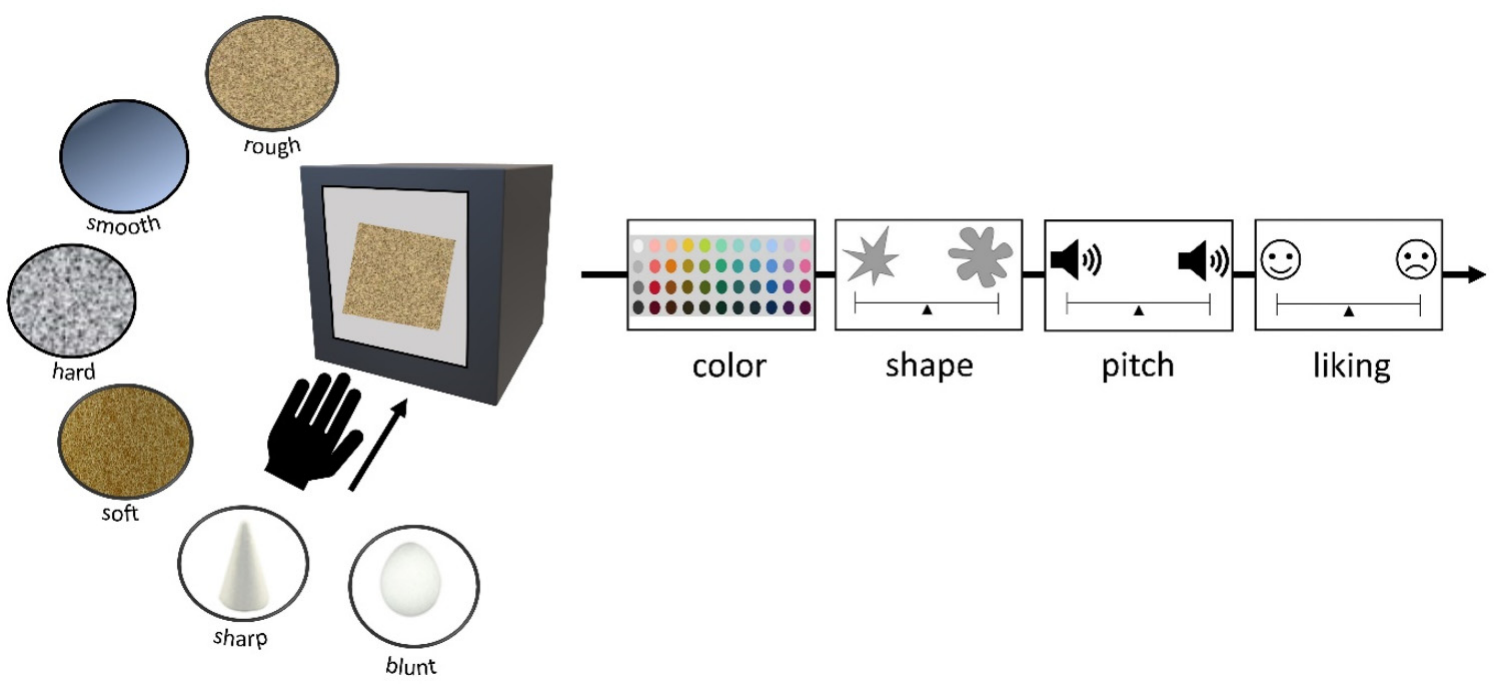
Schematic depiction of experiment 2. For each object participants chose a color, rated which shape and tone best matched the feel of the target object, and then rated how much they liked the feel of each object.

Participants were instructed to feel inside each opaque box one by one. Objects were not presented in pairs (e.g., hard and soft) but individually in a random order. The order of the to-be-rated stimuli (i.e., pitch, shape, and color) was fixed to avoid the task becoming too complicated for young children. For each object, they first selected a color match by clicking on the screen. They then heard each tone presented with a visual analog scale with a speaker icon on each end. Each tone was assigned to either the left or the right side of the scale. Participants were asked to indicate which sound goes with how the object feels by clicking on the scale where the object should be placed. They were next presented with the spiky and rounded shape, again with each shape assigned to either the left or the right side of the scale. Participants were asked to indicate which shape goes with how the object feels by clicking on the scale where the object should be placed. They were then asked to rate how much they liked the feel of the object by responding on a visual analog scale, with a happy face on one end of the scale and an unhappy face on the other end. Placement of all scale anchors was counterbalanced for left–right placement across participants. Location on the visual scales was translated into a score from 0 to 100. The task took around 5 min to complete. Figure 5 depicts the procedure.

### Results

Twenty-two participants were removed based on notes taken by the research assistants during testing: one participant was autistic (as stated by a parent), and the rest were removed due to problems with instructions or for sampling the boxes in an incorrect order (and therefore mismatching the experiment program). The remaining participants were divided into four age groups: 4–5 years (*n*  =  9), 6–9 years (*n*  =  93), 10–18 years (*n*  =  113), and 19 years and older (*n*  =  226). We first analyzed the data separately for each tactile object because we do not assume that choices are bipolar. For example, if participants significantly match the sharp object to a high pitch, it does not necessarily entail they would also match the blunt object to low pitch more than chance. We then compared mappings between each object in a pair (e.g., smooth versus rough). As a reminder, Ludwig and Simner (2013) found some associations between tactile properties in children and adolescents but not adults, and suggest associations for tactile properties are lost during development.

#### Pitch Judgments

**Figure 6.**
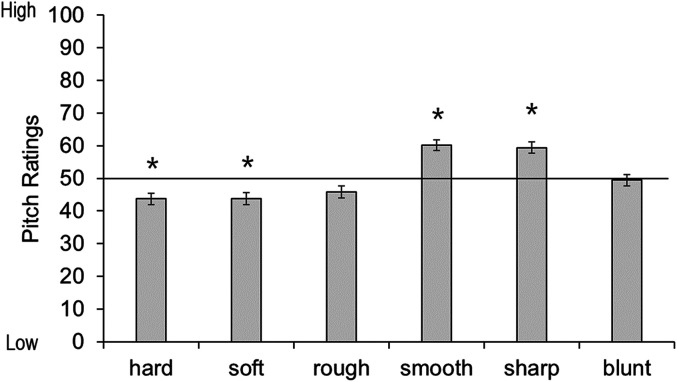
Mean pitch rating per tactile object. Ratings above 50 indicate a preference for high pitch, ratings below 50 indicate a preference for low pitch. Asterisks indicate a significant difference from the midpoint of the scale (corrected alpha *p* < .008). Error bars equal 1 *SE*.

Because the dependent variable is a scale from 0 to 100, one sample *t*-tests are appropriate to determine if ratings significantly differed from the middle of the scale (i.e., 50). One sample *t*-tests were conducted for each object across all participants with a Bonferroni-adjusted alpha based on the number of tests (i.e., 0.05/6  =  0.008). Sharp and smooth objects were significantly associated with high pitch: sharp *t*(441)  =  −6.11, *p* < .001, smooth *t*(441)  =  −6.66, *p* < .001; while hard and soft were significantly associated with low pitch: hard *t*(441)  =  4.08, *p* < .001, soft *t*(441)  =  3.92, *p* < .001. The blunt object and the rough object were not significantly associated with high or low pitch: blunt *t*(441)  =  0.38, *p*  =  .74; rough *t*(441)  =  2.52, *p*  =  .01 (see Figure 6).

**Figure 7.**
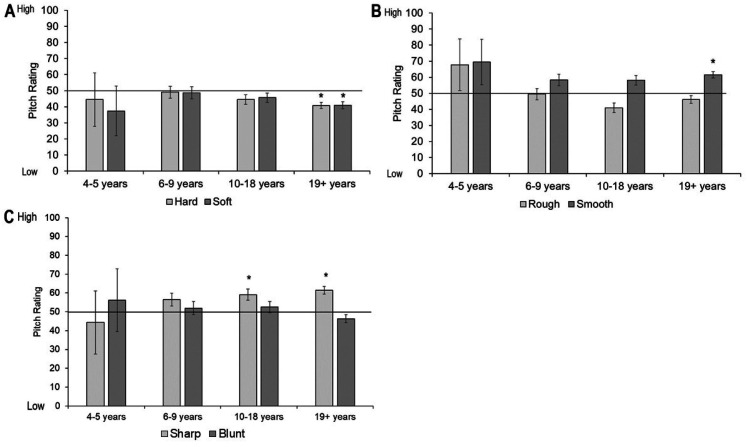
Pitch ratings across age groups for each tactile pair: (A) hard and soft, (B) rough and smooth, and (C) sharp and blunt. Ratings above 50 indicate a preference for high pitch, ratings below 50 indicate a preference for low pitch. Asterisks indicate a significant difference from the midpoint of the scale (corrected alpha *p* < .006). Error bars equal 1 *SE*.

To assess the effect of age, we conducted separate one sample *t*-tests for each object and each age group. For all objects, ratings of pitch matches for the 4- to 5-year-olds and 6- to 9-year-olds did not differ from chance. In 10- to 18-year-olds, the sharp object was matched to a high pitch, but none of the other ratings differed from chance. In the adult group, both hard and soft were significantly matched to the low pitch sound, smooth to a high pitch, and sharp to a high pitch. Ratings were not different from chance for the blunt or rough object (see Figure 7; corresponding statistics in Table B1 in Appendix B).

To compare pitch ratings between each object in a pair, we conducted three mixed analysis of variances (ANOVAs) with object as a within-participants variable (i.e., hard vs. soft, rough vs. smooth, and sharp vs. blunt) and age group a between-participants variable. There was no difference in pitch ratings between hard and soft objects, *F*(1, 437)  =  1.31, *p*  =  .25, η^2^_p_  =  0.003, and no interaction between object and age group, *F* < 1. There was no difference in pitch ratings between smooth and rough objects, *F*(1, 437)  =  3.05, *p*  =  .08, η^2^_p_  =  0.007, and no interaction between object and age group, *F* < 1. There was no difference in pitch rating between the sharp and blunt object, *F* < 1, and no interaction between object and age group, *F*(1, 437)  =  1.88, *p*  =  0.11, η^2^_p_  =  0.017. (See Supplemental Materials for analyses with age as a continuous predictor.)

#### Shape Judgments

**Figure 8.**
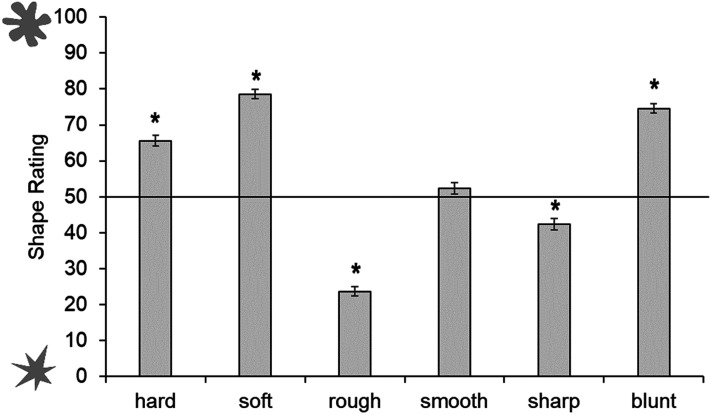
Mean shape rating for each tactile object. Ratings above 50 show a preference for rounded shape, ratings below 50 show a preference for spiky shape. Asterisks indicate a significant difference from the midpoint of the scale (corrected alpha *p* < .007). Error bars equal 1 *SE*.

One sample *t*-tests were conducted across all participants with a Bonferroni-adjusted alpha based on the number of tests (i.e., 0.05/6  =  0.008). Hard, soft, and blunt were significantly associated with the rounded shape: hard *t*(441)  =  10.55, *p* < .001, soft *t*(441)  =  21.73, *p* < .001, blunt *t*(441)  =  18.46, *p* < .001; whereas rough and sharp were associated with the spiky shape: rough *t*(441)  =  −19.00, *p* < .001, sharp *t*(441)  =  −4.89, *p* < .001. The smooth object was not significantly associated with the rounded or spiky shape, *t*(441)  =  1.38, *p*  =  .17 (see Figure 8).

**Figure 9.**
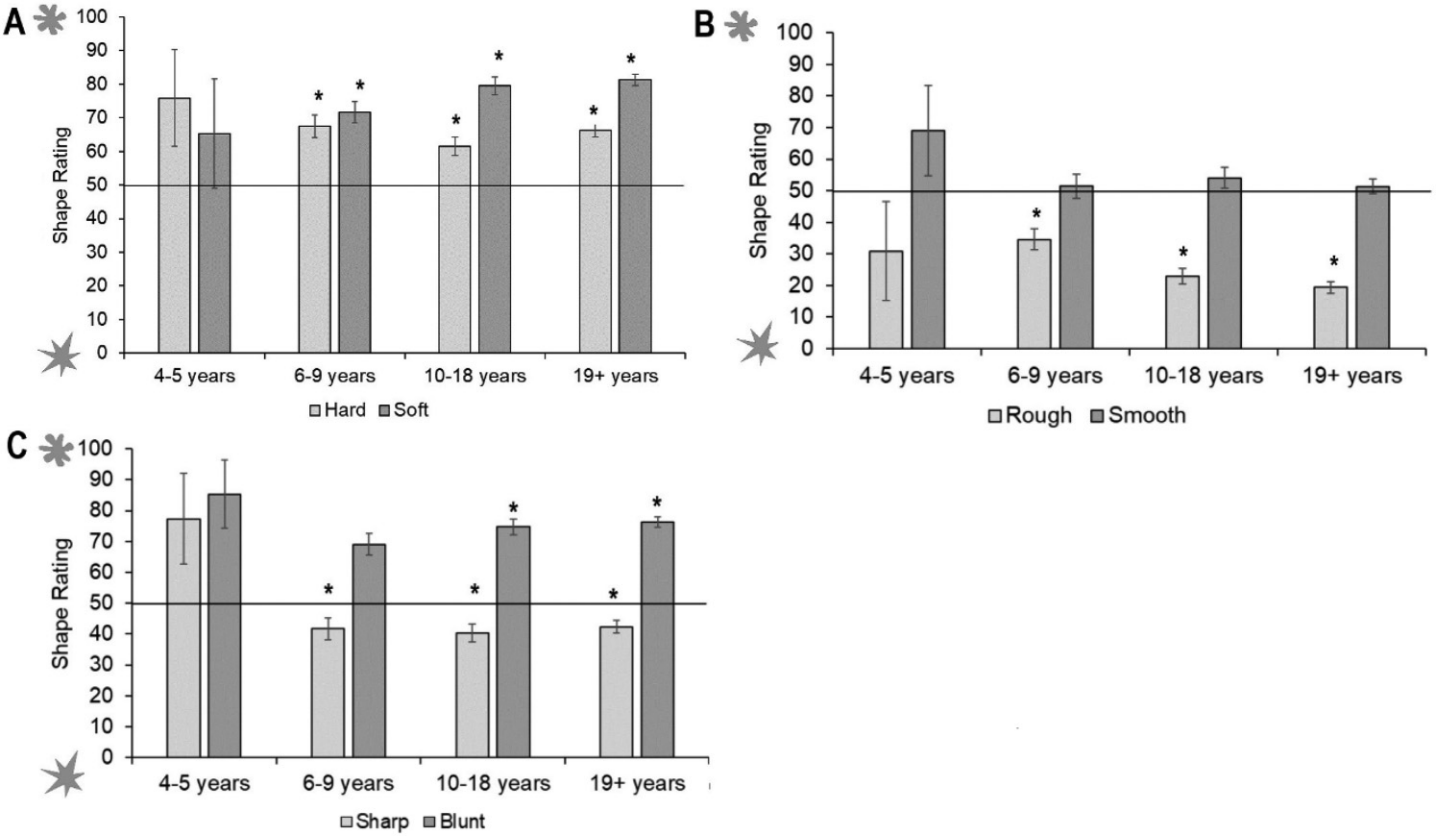
Shape ratings across age groups for each tactile pair: (A) hard and soft, (B) rough and smooth, and (C) sharp and blunt. Ratings above 50 show a preference for rounded shape, ratings below 50 show a preference for spiky shape. Asterisks indicate a significant difference from the midpoint of the scale (corrected alpha *p* < .006). Error bars equal 1 *SE*.

We then conducted separate one sample *t*-tests for each object and each age group. Four- to 5-year-olds showed no significant associations between the tactile property and visual shape. However, 6- to 9-year-olds associated hard, soft, and blunt objects to the rounded shape, and rough to the spiky shape. Ten- to 18-year-olds matched hard, soft, and blunt to rounded, and rough and sharp to the spiky shape. The adults showed the same pattern (see Figure 9; accompanying statistics in Table B1 in Appendix B).

To compare shape ratings between each object in a pair, we conducted three mixed ANOVAs with object as a within-participants variable (i.e., hard vs. soft, rough vs. smooth, and sharp vs. blunt) and age group as a between-participants variable. There was no difference in shape ratings between the hard and soft object, *F*(1, 437)  =  0.44, *p*  =  .51, η^2^_p_  =  0.001, but there was an interaction between object and age group, *F*(1, 437)  =  2.90, *p*  =  .02, η^2^_p_  =  .026. Follow-up pairwise comparisons found that the soft object was rated as rounder by the 10- to 18-year-olds, *t*(112)  =  4.86, *p* < .001, and those over 19-year-olds, *t*(225)  =  6.47, *p* < .001 only. The smooth object was rated as rounder than the rough object, *F*(1, 437)  =  5.76, *p*  =  .02, η^2^_p_  =  0.013, but there was no interaction between object and age group, *F*(1, 437)  =  2.08, *p*  =  .08, η^2^_p_  =  0.019. There was also a significant difference in shape rating between the sharp and blunt object, *F*(1, 437)  =  21.24, *p* < .001, η^2^_p_  =  0.046, with the blunt object rated as more rounded than the sharp object. There was no interaction between object and age group, *F*(1, 437)  =  1.92, *p*  =  .11, η^2^_p_  = 0 .017 (See Supplemental Materials for analyses with age as a continuous predictor).

#### Color Choices

Munsell colors were converted to CIE L*C*h^o^ values (following Ludwig & Simner, 2013). In this color space, L* describes color lightness (also known as luminance) from 0 (black) to 100 (maximum lightness/white), C* describes color chroma from 0 (unsaturated) to 100 (maximum chroma), and h^o^ refers to hue, reflecting 360° of hue that can be defined by hue categories (e.g., red, yellow, etc.). To ensure our analyses were comparable to that of Ludwig and Simner (2013), we compared chroma and lightness between pairs (hard–soft, rough–smooth, and sharp–blunt). Since the data did not follow a normal distribution, both chroma and lightness were analyzed with Wilcoxon signed ranks tests for each tactile pair in each age group. We used an adjusted alpha value of 0.0125 based on the number of tests for each tactile pair (i.e., 0.05/4  =  0.0125)

#### Chroma

**Figure 10.**
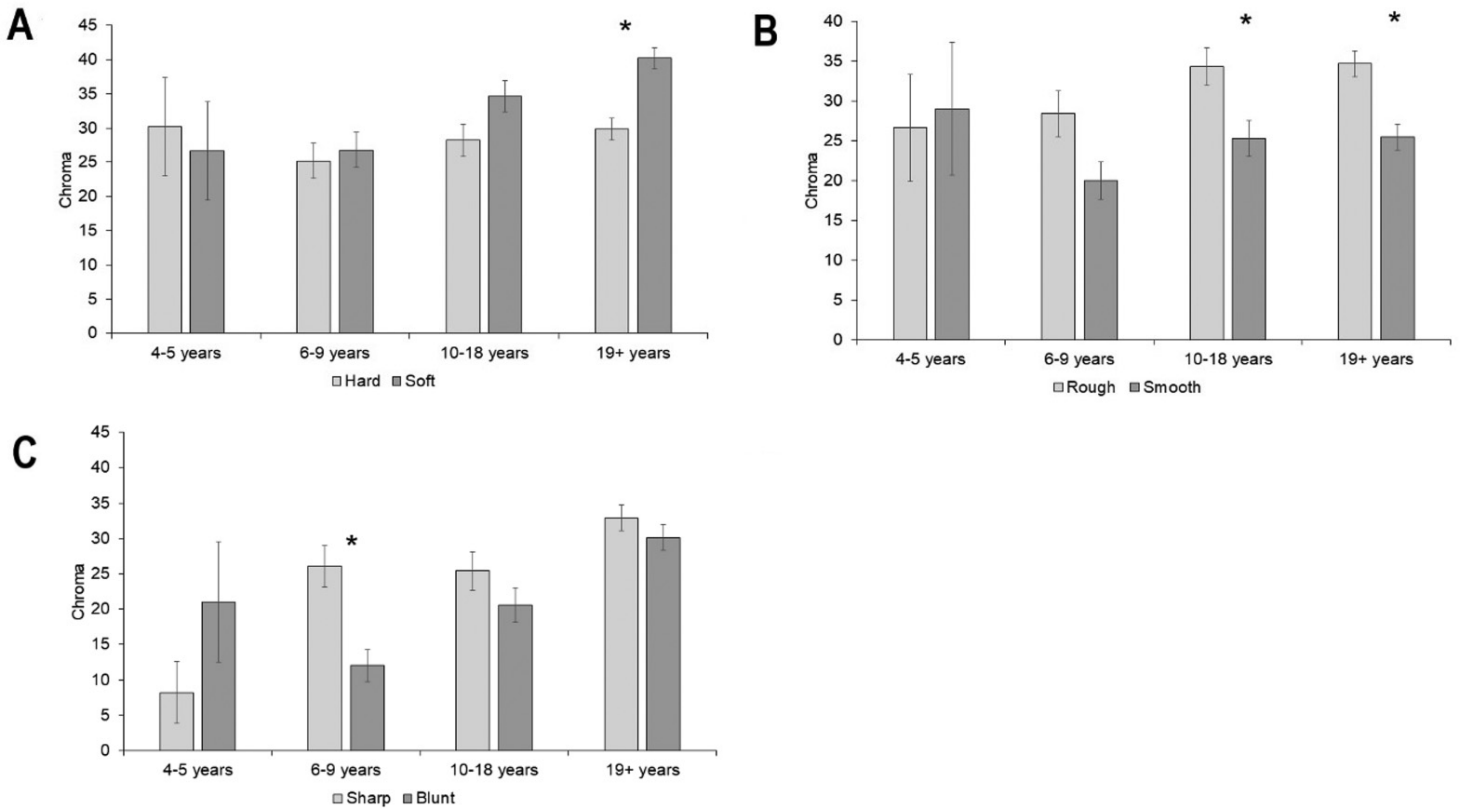
Mean chroma of color choices for each tactile pair: (A) hard and soft, (B) rough and smooth, and (C) sharp and blunt. Asterisk indicates a significant difference. Error bars equal to 1 *SE*.

Overall, colors matched to the soft object were higher in chroma than colors matched to the hard object, *z*  =  −4.66, *p* < .001. There was no difference in chroma between the hard and soft object for the 4- to 5-year-olds, *z*  =  .53, *p*  =  .59, 6- to 9-year-olds, *z*  =  −38, *p*  =  .71, and the 10- to 18-year-olds, *z*  =  −2.19, *p*  =  .03, but the 19+ group chose colors higher in chroma for the soft object compared to the hard object, *z*  =  −4.57, *p* < .001 Overall, colors chosen for the rough object were higher in chroma than colors chosen for the smooth object, *z*  =  −5.10, *p* < .001. There was no difference in chroma between rough and smooth for the 4- to 5-year-olds, *z*  =  0.28, *p*  = .78, or the 6- to 9-year-olds, *z*  =  −2.37, *p*  =  .02, but for the 10- to 18-year-olds, *z*  =  −2.96, *p*  =  .005, and the 19+ group, *z*  =  −3.48, *p*  =  .001, chroma was higher for the rough than the smooth object. Overall colors matched to the sharp object were higher in chroma than colors matched to the blunt object, *z*  =  −2.93, *p*  =  .003. The 6- to 9-year-olds chose colors with higher chroma for the sharp object than the blunt object, *z*  =  −3.59, *p* < .001, but there was no difference between sharp and blunt for the 4- to 5-year-olds, *z*  =  2.02, *p*  =  .04, 10- to 18-year-olds, *z*  =  −1.29, *p*  =  .19, or the 19+ group, *z*  =  −0.94, *p*  = .35 (see Figure 10; and Supplemental Materials for analyses with age as a continuous predictor).

#### Lightness

**Figure 11.**
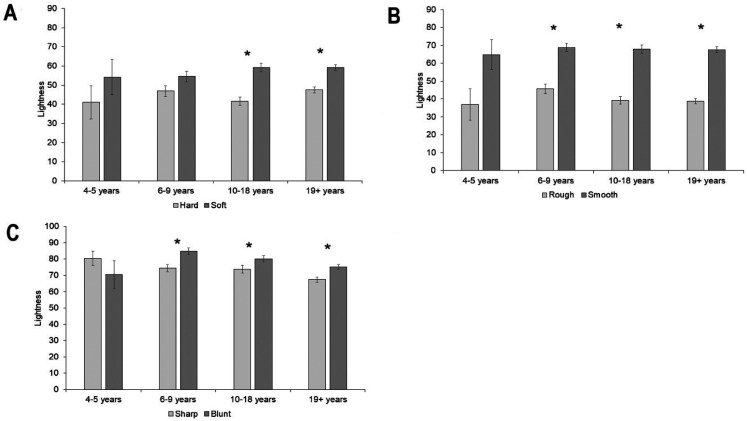
Mean lightness of color choices for each tactile pair: (A) hard and soft, (B) rough and smooth, and (C) sharp and blunt. Asterisk indicates a significant difference. Error bars equal to 1 *SE*.

Overall colors chosen for soft were lighter than those chosen for hard, *z*  =  7.47, *p* < .001. There was no difference in lightness between colors chosen for the hard and soft object for the 4- to 5-year-olds, *z*  =  1.1, *p*  = .31, or the 6- to 9-year-olds, *z*  =  2.05, *p*  =  .04. Colors chosen for soft were lighter than those chosen for hard in the 10- to 18-year-olds, *z*  =  4.86, *p* < .001, and the adults, *z*  =  5.46, *p* < .001. Overall, colors matched to the smooth object were lighter than colors matched to the rough object, *z*  =  13.79, *p* < .001. There was no difference in lightness between the rough and smooth objects for the 4- to 5-year-olds, *z*  =  2.1, *p*  =  .04, but colors chosen for the smooth object were lighter than those chosen for the rough object for the 6- to 9-year-olds, *z*  =  4.85, *p* < .001, the 10- to 18-year-olds, *z*  =  7.1, *p* < .001, and the adults, *z*  =  10.87, *p* < .001. Overall colors chosen for the blunt object were lighter than those chosen for the sharp object, *z*  =  5.96, *p* < .001. There was no difference in lightness between the sharp and blunt objects for the 4- to 5-year-olds, *z*  =  −0.94, *p*  =  .35, but colors chosen for the blunt object were lighter than those chosen for the sharp object for the 6- to 9-year-olds, *z*  =  3.84, *p* < .001, the 10- to 18-year-olds, *z*  =  2.88, *p*  =  .004, and the adults, *z*  =  4.06, *p* < .001 (see Figure 11 and Supplemental Materials for analyses with age as a continuous predictor).

#### Hue

**Figure 12.**
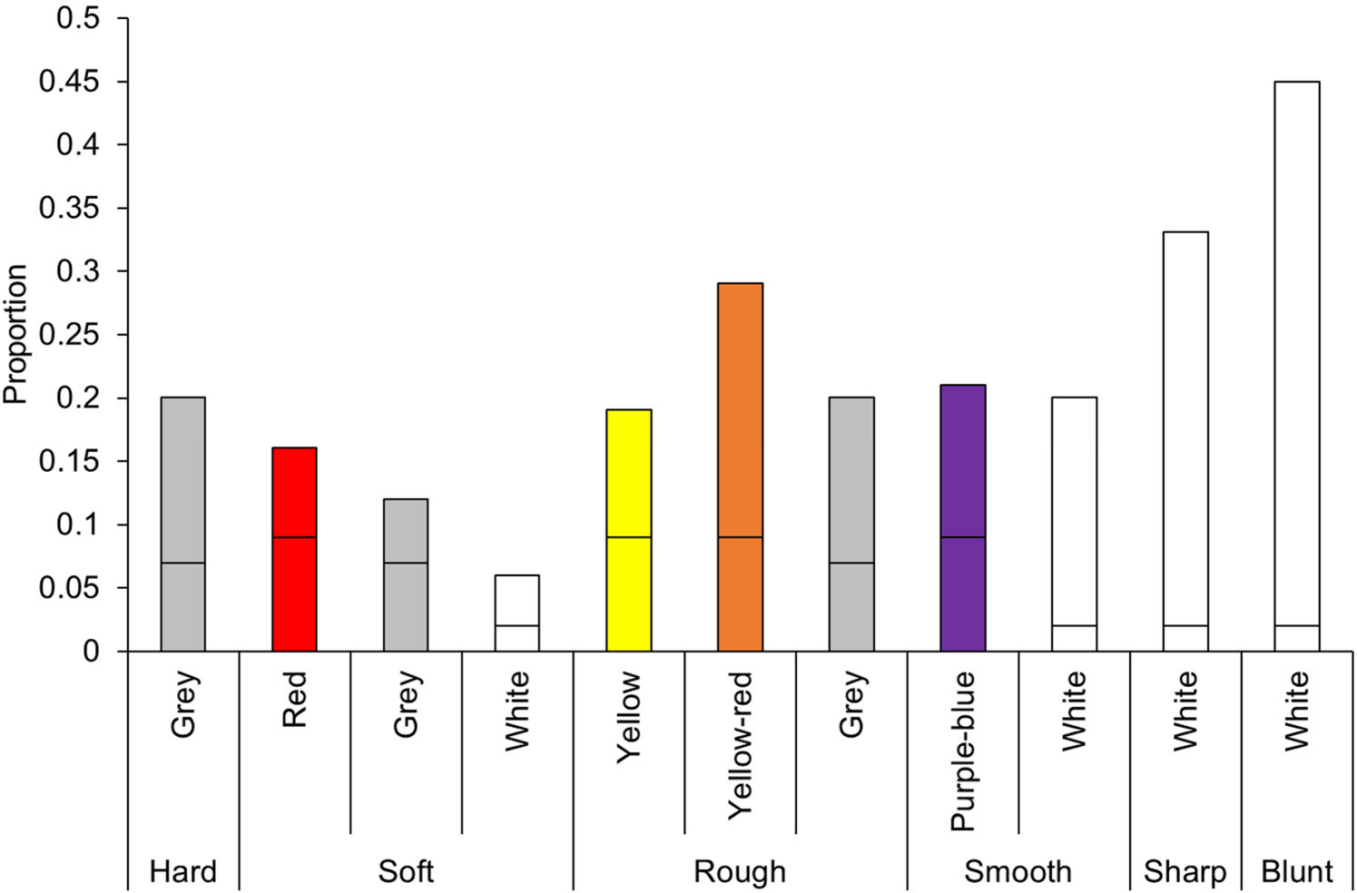
Proportion of hue choices selected at greater than chance level for each object. Horizontal lines on the bars indicate chance levels for each hue. Chance level differs by hue because the number of color chips per hue differed. Colors do not reflect actual color chips used.

For each tactile object, the total number of color choices per hue was calculated. To determine whether the number of choices of each color differed from chance, a binomial test for each hue and each object was appropriate. Chance level differed by hue because the number of color chips per hue was not equal. For example, there were four color chips with a blue–green hue and only one white color chip. The probability of choosing a blue–green chip would be 4 out of 44 color chips (.09), whereas the probability of choosing a white color chip would be 1 out of 44 (0.02). A Bonferroni-corrected alpha of *p* < .004 was used based on the number of colors tested for each object (0.05 divided by 12 colors; see Figure 12).

The hard object was significantly matched to grey, *z*  =  11.85, *p* < .001. Soft was significantly matched to red, *z*  =  5.49, *p* < .001, grey *z*  =  3.48, *p* < .001, and white, *z*  =  6.06, *p* < .001. The rough object was significantly matched to yellow, *z*  =  7.60, *p* < .001, yellow–red, *z*  =  14.91, *p* < .001, and grey, *z*  =  10.73, *p* < .001. The smooth object was significantly matched to purple–blue, *z*  =  9.10, *p* < .001, grey, *z*  =  8.87, *p* < .001, and white, *z*  =  26.39, *p* < .001. The sharp object was matched to white, *z* =  45.75, *p* < .001, but the blunt object was also matched to white, *z*  =  64.10, *p* < .001.

We then conducted binomial tests separately for each object and age group. For 4- to 5-year-olds, there were no significant color matches for the hard, soft, rough, smooth, or sharp object, but the blunt object was matched to white more than chance. For 6- to 9-year-olds, hard and soft were matched to grey, rough to grey and yellow, and smooth to grey and white, while both sharp and blunt were matched to white. Ten- to 18-year-olds, matched hard to grey, whereas soft was matched to white; rough was matched to grey, yellow, and yellow–red; smooth was matched to purple–blue, grey, and white; and both sharp and blunt were matched to white. Adults (from 19 onwards) matched hard to grey; soft to red; rough to grey, yellow, and yellow-red; smooth to purple–blue, grey, and white; and both sharp and blunt to white (see Table B2 in Appendix B for details). See Supplemental Materials for analyses with age as a continuous predictor.

### Summary

Overall we found sharp and smooth objects were matched to high pitch sound, but objects with the opposite tactile properties (i.e., blunt and rough) were not significantly associated with pitch. This suggests these mappings are unipolar and not bipolar. Both hard and soft objects were matched to low pitch sound, but it is possible that choices were made based on shape instead of hardness since the shape of the objects was the same. Although the same pattern of associations was observed for the adult group, no associations emerged in the other groups, except between the sharp object and high pitch in the 10- to 18-year-olds.

Most objects were associated with a shape. The blunt object was matched to the rounded shape and the sharp object to the spiky shape. Both hard and soft objects were matched to the rounded shape. Again, this is likely due to both stimuli having a somewhat rounded shape (i.e., a tube). Rough was matched to the spiky shape, but there was no association between smooth and specific shapes. No associations were observed in the youngest participant group, but in the older groups patterns of association were broadly the same.

Overall colors matched to the soft object were higher in chroma than the hard object, but this pattern was only observed in the oldest age group. This is at odds with the results of Ludwig and Simner (2013) who found colors matched with soft were higher in chroma for children and adolescents, but not adults. Colors matched to the rough object were higher in chroma than those matched to the smooth object, but this pattern was only present in the 10- to 18-year-olds and adults. This also goes against the results of Ludwig and Simner (2013) who found an effect of smoothness on chroma in 5- to 9-year-olds, with the same trend in 10- to 18-year-olds, but found no effect in older groups. Furthermore, the observed differences between rough and smooth are also opposite to those reported in Ludwig and Simner (2013). We found rough was matched to colors with higher chroma than smooth, whereas Ludwig and Simner (2013) found the opposite with smooth matched to colors with higher chroma than rough. Colors matched to the sharp object were higher in chroma than the blunt object, but this was only the case for the 6- to 9-year-olds.

The colors chosen were lighter for the soft object than the hard object, smooth over rough, and blunt over sharp. This is in line with Ludwig and Simner (2013) who found objects that were smoother, softer, and rounder were higher in lightness across all ages. These patterns were not observed in the youngest age group, however. All objects had significant associations with hues, but the 4- to 5-year-olds only showed a significant match for the blunt shape.

Overall, our results belie the finding that tactile–color associations occur in children but not adults (Ludwig & Simner, 2013). Instead, the youngest age group displayed almost no associations with tactile properties. More generally, a greater number of associations emerged in the older participant groups, suggesting learned associations from the environment.

## Experiment 3: Odor

### Method

#### Participants

Four-hundred and fifty-six participants volunteered to take part in the experiment (242 females, 3 participants’ gender was not recorded, 1 participant's age was not recorded, age *M*  =  25.95 years, *SD*  =  18.27, range  =  4–81 years). All other details were as before.

#### Materials

Five Sniffin’ sticks with the odors of caramel, lemon, onion, menthol, and raspberry were utilized, taken from the Burghart standard identification test. Odors were selected based on reported crossmodal associations with these odors from previous studies. The same color, shape, and pitch stimuli from Experiment 2 were used, as well as a piece of sandpaper (rough) and plastic (smooth) as possible tactile matches for odors.

#### Procedure

**Figure 13.**
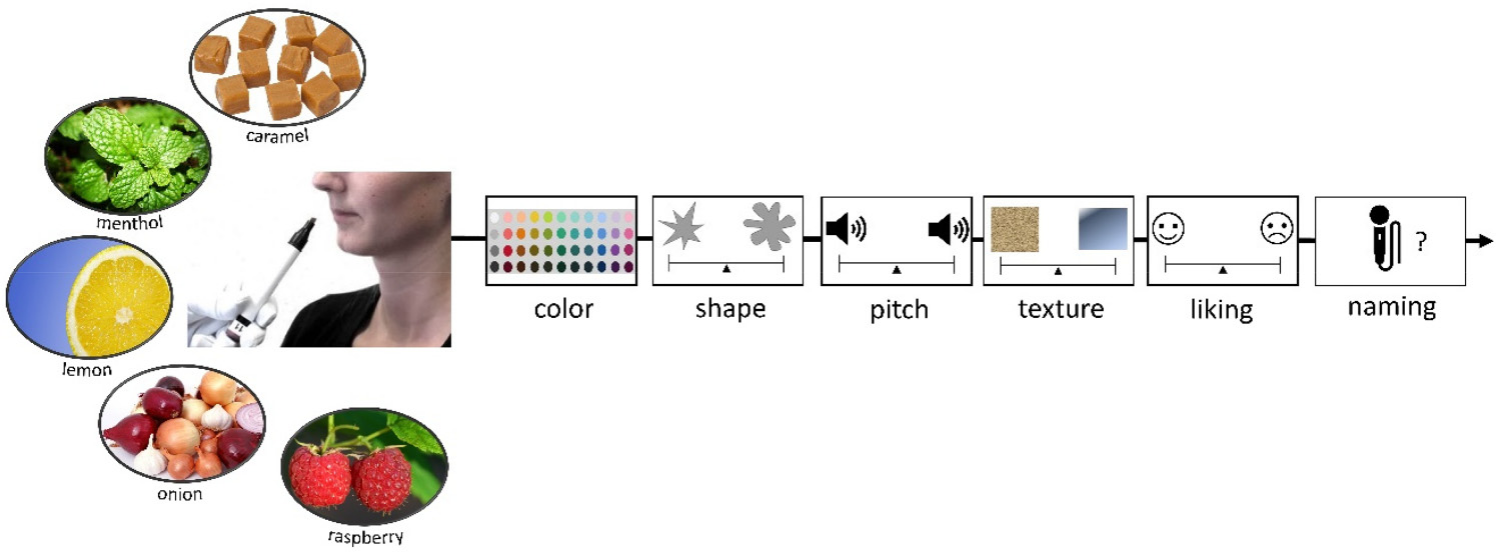
Schematic depiction of experiment 3. For each odor, participants chose a color and then rated what shape, tone, and tactile property best matched that odor, and how much they liked the odor. They were then asked to name the odor.

Participants were presented with one odor at a time and had to select a color and make ratings of pitch and shape as described in Experiment 2. Participants were not told the names of the odors. For odor-to-tactile judgments, participants were instructed to feel inside two boxes, and rate which tactile property matched the odor by clicking on a visual scale. The placement of tactile boxes was fixed to reduce errors in the data due to the fact that the study was administered by a research assistant by hand rather than an experimental program. After tactile matches, participants were asked to rate how much they liked each odor by responding on a visual analog scale, with a happy face on one end and a sad face on the other end. Location on the scale was measured on a 0 to 100 scale. Finally, each participant was asked to name the odor by typing their response. An experimenter typed in responses for children who were not able to type or spell by themselves. The whole task took around 5 min. The procedure is depicted in Figure 13.

### Results

Six participants were removed from analysis based on notes taken by the research assistants during testing: one for chewing gum during the task, one had a cold, three did not follow instructions, and one participant was autistic (as stated by a parent). The remaining participants were divided into four age groups: 4–5 years (*n*  =  13), 6–9 years (*n*  =  89), 10–18 years (*n*  =  111), and 19 years and older (*n*  =  236). To assess whether ratings were significantly different from the midpoint of the rating scale, separate one-sample *t*-tests were conducted for each odor and dimension. The alpha level was adjusted based on the number of tests conducted per odor and dimension (i.e., 0.05/4  =  0.0125).

#### Pitch Ratings

**Figure 14.**
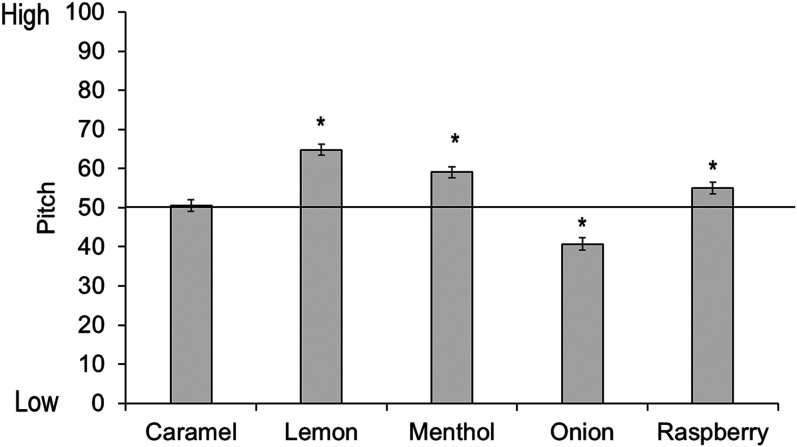
Mean pitch ratings per odor. Ratings above 50 show a preference for high pitch, ratings below 50 show a preference for low pitch. Asterisks indicate a significant difference from the midpoint of the scale (corrected alpha *p* < .01). Error bars equal 1 *SE*.

There were no reliable associations between caramel odor and pitch, *t*(449)  =  0.36, *p*  =  .72, but lemon *t*(449)  =  10.16, *p* < .001, menthol *t*(449)  =  6.47, *p* < .001, and raspberry *t*(449)  =  3.34, *p* < .001 were all significantly associated with high pitch, while onion was significantly associated with low pitch, *t*(449)  =  −5.87, *p* < .001 (see Figure 14).

**Figure 15.**
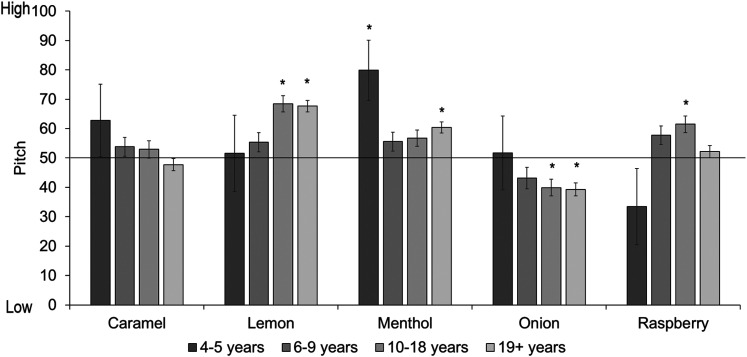
Mean pitch ratings per odor and age group. Ratings above 50 show a preference for high pitch, ratings below 50 show a preference for low pitch. Asterisks indicate a significant difference from the midpoint of the scale (corrected alpha *p* < .01). Error bars equal 1 *SE*.

We then conducted tests separately by age group (see Figure 15). For the youngest children, menthol was significantly matched to a high pitch, but there were no other reliable associations. For 6- to 9-year-olds there were no significant effects. Ten- to 18-year-olds matched lemon and raspberry odors to a high pitch, and onion to a low pitch, while adults matched lemon and menthol odors to a high pitch, and onion to a low pitch. (Full statistics can be found in Table C1 in Appendix C; analyses with age as a continuous predictor can be found in Supplemental Materials.)

#### Shape Ratings

**Figure 16.**
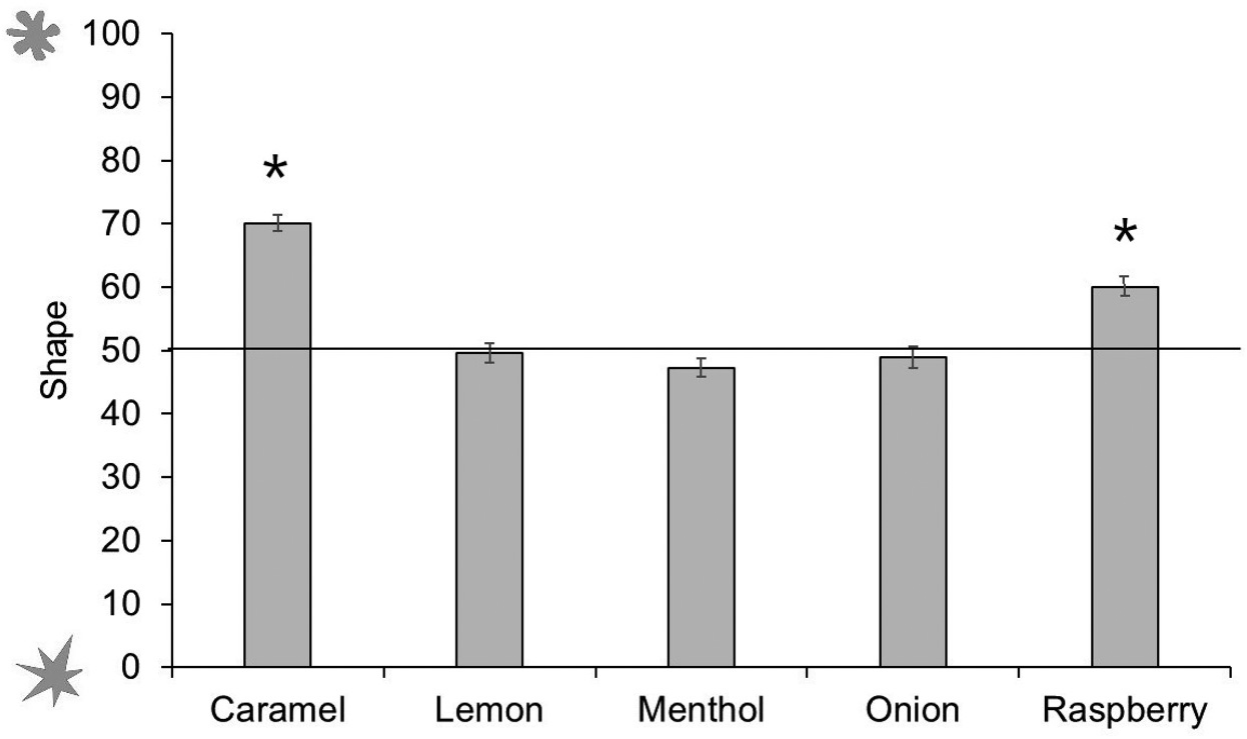
Shape ratings across odors. Ratings above 50 show a preference for rounded shape, ratings below 50 show a preference for spiky shape. Asterisks indicate a significant difference from the midpoint of the scale (corrected alpha *p* < .0125). Error bars equal 1 *SE*.

Analyses were conducted in the same manner as pitch ratings. Caramel and raspberry odors were significantly associated with rounded shape, *t*(449)  =  6.94, *p* < .001. There were no other reliable associations between odors and shape: lemon odor *t*(449)  =  −0.28, *p*  =  .78; menthol odor *t*(449)  =  −1.84, *p*  =  .07; onion odor *t*(449)  =  −0.63, *p*  =  .53 (see Figure 16).

**Figure 17.**
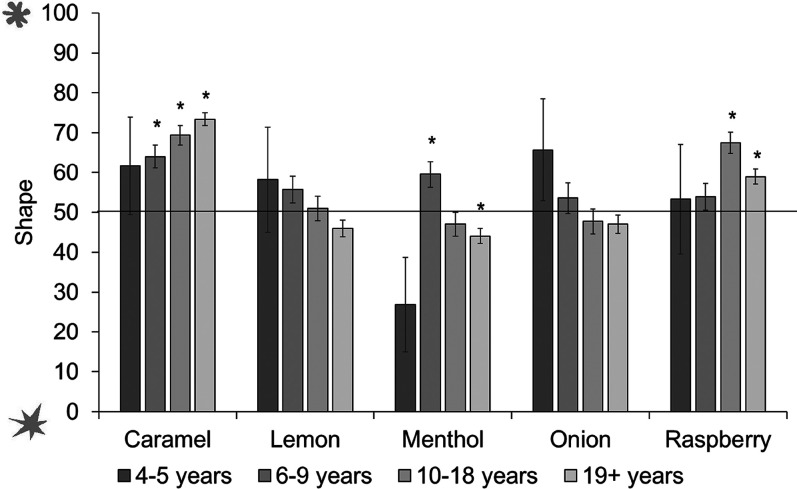
Shape ratings across odors and age groups. Ratings above 50 show a preference for rounded shape, ratings below 50 show a preference for spiky shape. Asterisks indicate a significant difference from the midpoint of the scale (corrected alpha *p* < .0125). Error bars equal 1 *SE*.

We then conducted separate tests by age group (see Figure 17). The youngest children did not associate any odors reliably with spiky or rounded shapes. Six- to 9-year-olds matched caramel and menthol to the rounded shape; 10- to 18-year-olds also matched caramel, as well as raspberry, to the rounded shape; while adults matched caramel and raspberry to the rounded shape, and menthol to the spiky shape. (Analyses with age as a continuous predictor can be found in Supplemental Materials.)

#### Tactile Ratings

**Figure 18.**
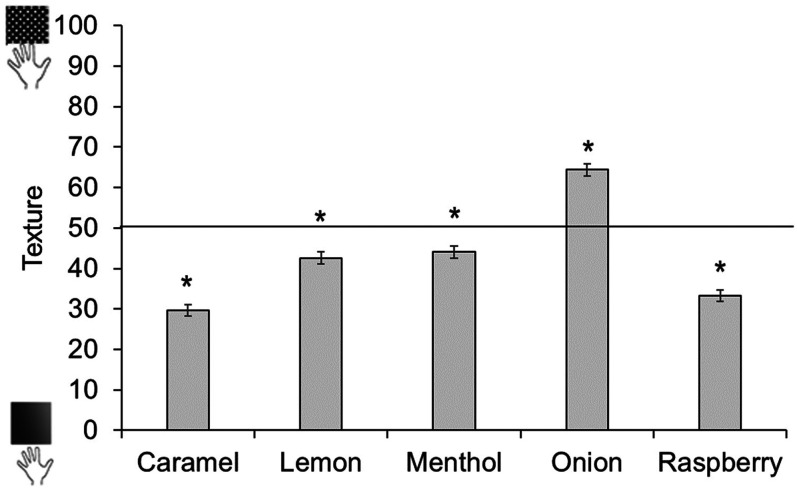
Tactile ratings across odors. Ratings above 50 show a preference for rough, ratings below 50 show a preference for smooth. Asterisks indicate a significant difference from the midpoint of the scale (corrected alpha *p* < .0125). Error bars equal 1 *SE*.

Tactile ratings were analyzed in the same way as pitch and shape ratings. Onion odor was significantly associated with rough, *t*(454)  =  9.49, *p* < .001, whereas all other odors were significantly associated with smooth: caramel *t*(454)  =  −14.72, *p* < .001; lemon *t*(454)  =  −4.63, *p* < .001; menthol *t*(453)  =  −3.79, *p* < .001; raspberry *t*(454)  =  −11.99, *p* < .001 (see Figure 18).

**Figure 19.**
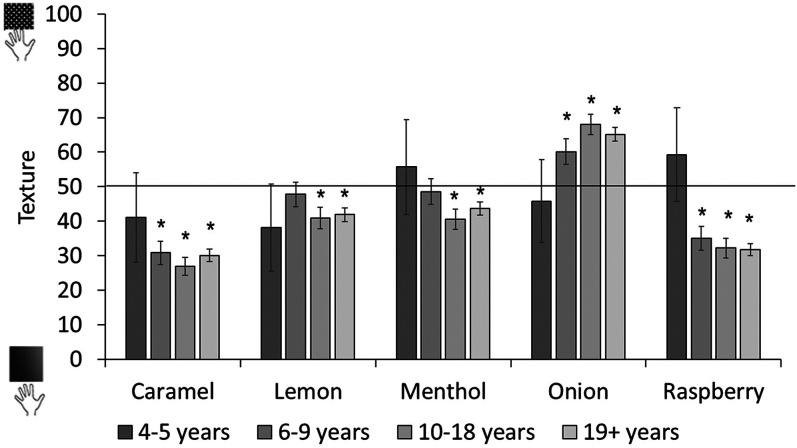
Tactile ratings across odors and age groups. Ratings above 50 show a preference for rough, ratings below 50 show a preference for smooth. Asterisks indicate a significant difference from the midpoint of the scale (corrected alpha *p* < .0125). Error bars equal 1 *SE*.

We then analyzed tactile ratings separately by age group (see Figure 19). The youngest children showed no evidence of reliable odor–tactile associations. Six- to 9-year-olds associated caramel and raspberry to smooth, and onion to rough; while 10- to 18-year-olds matched caramel, lemon, menthol, and raspberry to smooth, and onion to rough. The same pattern was observed in adults. (Analyses with age as a continuous predictor can be found in Supplemental Materials.)

**Figure 20.**
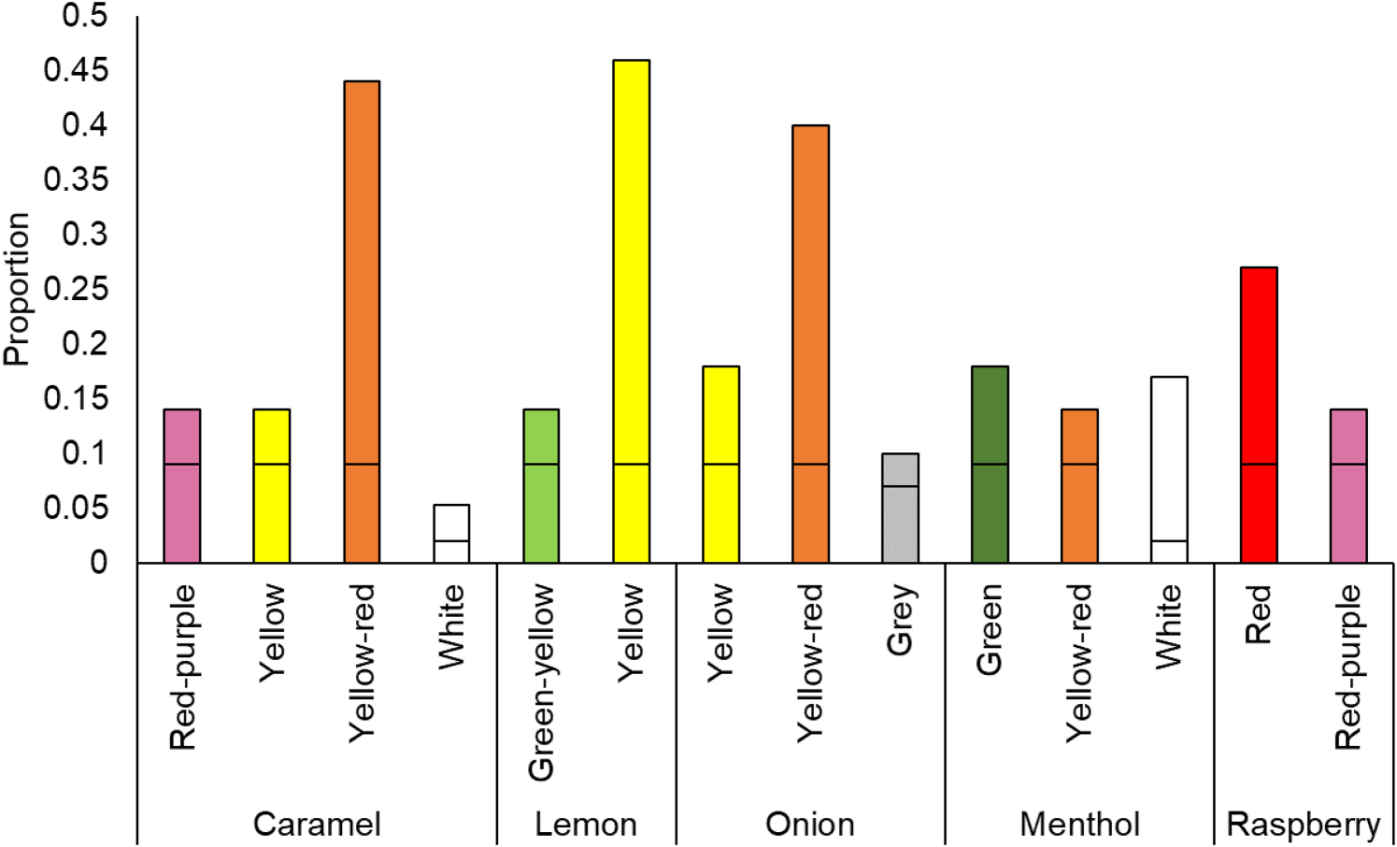
Proportion of hue choices per odor for significant colors. Horizontal lines on the bars indicate chance levels for each hue. Chance level differs by hue because the number of color chips per hue differed.

#### Color Choices

##### Hue

Hue choices were analyzed in the same way as the tactile–hue data with a binomial test for each odor to determine whether the number of color choices for each odor differed from chance, again with a Bonferroni-adjusted *p*-value of .004, based on the number of colors tested for each object (0.05 divided by 12 colors; see the “Hue” section for details).

Caramel odor was significantly matched to red–purple, *z*  =  3.29, *p* < .001, yellow, *z*  =  3.46, *p* < .001, yellow-red, *z*  =  25.70, *p* < .001, and white, *z*  =  4.14, *p* < .001. The lemon odor was significantly matched to green–yellow, *z*  =  3.29, *p* < .001, and yellow, *z*  =  27.18, *p* < .001.

Onion odor was significantly matched to yellow, *z*  =  6.59, *p* < .001, yellow-red, *z*  =  23.23, *p* < .001, and grey, *z*  =  2.98, *p*  =  .001. Menthol odor was significantly matched to green, *z*  =  6.78, *p* < .001, yellow–red, *z*  =  3.48, *p* < .001, and white, *z*  =  20.52, *p* < .001. Finally, raspberry odor was matched significantly to red, *z*  =  13.51, *p* < .001, and red–purple, *z*  =  3.46, *p* < .001 (see Figure 17).

We then conducted the same analyses separately by odor and age group. Four- to 5-year-olds matched caramel odor to yellow–red, lemon to yellow, and menthol to white. There were no significant matches to raspberry or onion. Six- to 9-year-olds matched caramel odor to yellow–red, like the younger group, but also to red–purple. The lemon odor was matched to yellow, onion to yellow and yellow–red, menthol to white, and raspberry to red. Ten- to 18-year-olds matched caramel to yellow-red and white, lemon to yellow, onion to yellow and yellow–red, menthol to white, and raspberry to red and red–purple. Adults also matched caramel to yellow and yellow–red, lemon to yellow and green–yellow, onion to yellow–red, menthol to green and white, and raspberry to red. (Results are shown in Table C2 in Appendix C. Analyses with age as a continuous predictor can be found in Supplemental Materials.)

### Summary

The present study did not replicate an association between caramel odor and low pitch (Belkin et al., 1997) but did support the previously observed association between fruity odors (lemon and raspberry) and high pitch (Crisinel & Spence, 2012a). There was also an association between menthol odor and high pitch, and between onion odor and low pitch. We did not replicate an association between lemon odor and spiky shape (Hanson-Vaux et al., 2013), which could be due to differences in stimuli instantiation: because we used a Sniffin’ Sticks odorant instead of a real lemon. An association between raspberry odor and the rounded shape was however replicated (Hanson-Vaux et al., 2013). We also found associations between smoothness and lemon odor, and roughness and onion odor, previously observed using texture words (Spector & Maurer, 2012). However, in contrast to Spector and Maurer (2012), we found menthol was matched to smooth and not rough texture.

For odor–pitch associations, children under age 6 showed one significant mapping: menthol was matched to the high pitch tone, and this was also evident in those over 19-year-olds. However, no matches were observed for children between 6- and 9-year-olds. From age 10 into adulthood, lemon was matched to high pitch and onion to low pitch. Raspberry was also matched to high pitch in the 10- to 18-year-olds. Caramel odor showed no mappings with pitch.

Odor–shape associations were not observed in children under age 6 years. In all other groups, caramel odor was matched to the round shape. Raspberry was matched to the rounded shape only from 11 years old; menthol to the rounded shape in the 6- to 9-year-olds, but to the spiky shape in the over 19-year-olds group; and lemon to spiky only in those over 19 years old. Onion was the only odor not matched to a shape.

As with odor–shape associations, children under 6 years old did not show any mappings between odor and tactile properties. Associations between odor and tactile properties emerged from age 6, with the largest number of associations occurring after age 10. In participants over 10 years old, all odors were rated as smooth, except for onion which was rated as rough.

Overall odors tended to be matched to the colors of their sources. As with the other dimensions, the strength of associations appeared to increase with age, with more odors being matched to colors generally. The youngest age group did not show any significant color matches to raspberry odor, consistent with the lack of mappings between this odor and shape, pitch, and tactile properties. In general, associations with odors seem to be consistent from age 10.

## Discussion

This study provides support for associations between the senses, replicating a number of previously observed crossmodal associations in a noisier setting outside of the lab with a diverse participant sample. We also provide new insights into the development of crossmodal associations involving sound, tactile properties, and the least-explored sense—smell. For the first time, we systematically examined a range of associations in children, adolescents, and adults, and found few associations appeared in early childhood; instead, associations arise and become more robust into adulthood.

Our results suggest that associations change over time through experience. One possible form of experience is learning statistical associations from the environment (Spence, 2011). For example, objects found in high spaces typically emit higher pitch sounds, and certain odors are emitted from objects with a specific color (e.g., lemon–yellow). An alternative route to learning crossmodal associations is from semantic information, specifically through language. For example, the increased mapping between high pitch and high space is enhanced when high pitches are described as *high* in the language (Dolscheid et al., 2020). Language may also serve to reinforce associations learned from the environment: for instance, odor–color associations differ depending on how odors are described (de Valk et al., 2017; Goubet et al., 2018; Speed & Majid, 2018), and the mapping between thickness and pitch is more robust in young children who are learning a language that uses a thickness metaphor to describe pitch (Shayan et al., 2014). Words may therefore play a crucial role in the way information from multiple senses is bound together. Finally, changes over time could also reflect developmental changes in perception, for example, maturation of supramodal size perception (Cuturi et al., 2019).

Another possibility to consider is that crossmodal associations are mediated by emotion, as has been shown for sound and color (Palmer et al., 2013), and music and flavor (Crisinel & Spence, 2012b). This would be an example of what Walker et al. (2020) call *mediated* associations. Emotional congruency could have driven some associations in the present tasks (see Deroy et al., 2013): unpleasant colors (e.g., brown) were matched to unpleasant odors (e.g., onion) and unpleasant tactile properties (e.g., rough). Pleasantness ratings we collected show that onion was the only odor considered unpleasant and rough was the only tactile property considered unpleasant.

One set of associations that is more difficult to explain in terms of the mechanisms outlined above is the association between tactile properties and hue. These associations cannot be accounted for by a statistical explanation, since tactile properties can belong to objects of any color, although we note that both sharp and blunt objects were matched to white, which is likely to do with the specific material of the object (polystyrene). Some associations (e.g., soft and red hue) are likely to reflect the Western association between softness and pink (e.g., the phrase *soft pink*; Ludwig & Simner, 2013). However, it has been reported that monkeys associate round, soft, and pink too (Premack & Premack, 2003; as cited in Spence & Deroy, 2012), suggesting the association could be nonlinguistic and possibly innate (although no independent evidence has been found for this, Spence & Deroy, 2012). Alternatively, tactile–color matches could be mediated by emotional associations, as suggested above. In general, the mappings that we observed across the studies are likely to be explained by some combination of the mechanisms above, but further exploration is needed to disentangle the possible contributions of each.

Previously, Ludwig and Simner (2013) found tactile–color associations in children but not adults. On the contrary, we found that colors associated with softness increased in chroma with age. Our results also diverged from Ludwig and Simner (2013) in that we found associations between smoothness and chroma were not affected by age. The differences between the two studies could reside in the specific methods utilized. For example, we tested a single stimulus for each tactile property (i.e., one smooth object and one rough object), but Ludwig and Simner (2013) used a continuum (i.e., surfaces differed gradually from rough to smooth in six steps). One consequence of having multiple stimuli on a continuum is that they cannot be easily labeled and compared. That is, one surface cannot be easily categorized as the “smooth” surface. Labeling stimuli could lead to color choices through the semantic system (e.g., the rough stimulus receives a color that is high in chroma, and so the smooth stimulus receives a low chroma, because it is its opposite). This tactic of labeling and comparing is likely to occur more in adults since they have stronger language and memory skills (Gentner, 2016). However, labeling stimuli would be more difficult when presented with a continuum. Contrary to this possibility, some tactile associations observed in Experiment 2 were not symmetrical (e.g., sharp was matched to a high pitch, but blunt was not matched to a low pitch), suggesting adult choices are not always based on antonymic labels. Another possibility is that multiple exemplars of a perceptual attribute may make dimensions more salient. Perhaps children in our study did not hone in on the relevant perceptual feature, but would have with more exemplars. Future studies could systematically manipulate such experimental features to uncover the mechanisms underlying tactile–chroma associations.

This study is the first to assess multiple crossmodal associations with odor across development using perceptual stimuli. Associations between odor and pitch, odor and shape, and odor and tactile properties were more likely to be observed in older age groups. In contrast, we observed evidence for some odor–color associations changing over development. For example, choices of green for menthol odor and yellow–green for lemon odor occurred only in the adult group. This is in line with previous evidence that suggests odor–color associations are flexible throughout development depending on factors such as semantic and lexical knowledge, and environmental or cultural experience (Goubet et al., 2018). At a young age, children may associate the odor of menthol with the color of mint products such as their toothpaste (white) but later they learn that the odor comes from the green mint plant. Contrary to what might have been expected given the unique status of olfaction (see Introduction), odor does not appear to be distinct in its patterns of associations in comparison to pitch and tactile properties, where associations also increase with age. Odor is therefore likely to also be a relevant modality in multisensory interaction (cf. Deroy et al., 2013).

Since our tasks were designed to be quick and simple to administer in a museum setting, we were limited in the number of variables that could be measured. Future studies should build upon these initial results and assess how certain factors, such as language and emotion, may affect the development of specific crossmodal associations. Furthermore, since we collected data from an opportunistic sample, we had little control over the variability in the age of our participants. Ideally, future studies would match the number of participants across age groups and therefore be able to assess which time points are critical in the development of crossmodal associations. Additionally, a longitudinal study rather than a cross-sectional study would provide stronger support for a developmental effect of experience.

Although there was a wide age range in the current study, only 6% of participants were over 60 years of age. This is likely to mean that our data was not sensitive enough to detect any differences in associations that may occur with aging (Freiherr et al., 2013). Related to this, since our youngest participant was four years old, we cannot make claims regarding crossmodal associations before this age. Previously, some authors have suggested some crossmodal associations follow a U-shaped function (e.g., Maurer & Maurer, 1988; Maurer & Mondloch, 2005), where associations that are observed early, are then lost, only to reappear at an older age. With the present data, we cannot rule out learning before age four.

There may also be some practical limitations to these studies, since they were conducted inside a science museum, which is an exciting and stimulating place for a child. Factors such as stress and task demands may affect how a sensory task is conducted more in children than adults (Lavin & Lawless, 1998). On the other hand, testing took place in a quiet room away from the main museum hall, with experimental conditions adapted to the circumstances. Furthermore, the tasks were designed to be short and simple so they could be completed by children quickly and easily. Despite adequate control of distractions and a simple task, time constraints meant that we could only test a limited number of stimuli. Nevertheless, the data presented here provide a strong basis for planning future controlled laboratory studies with a larger number of stimuli.

## Conclusion

Using a large number of participants across a wide age range, we found evidence for crossmodal associations with pitch, tactile properties, and odor, replicating associations previously observed in smaller, focused studies. In general, we find crossmodal associations emerge over time. This points to the importance of statistical associations, as well as language and semantics in the development of crossmodal associations. Future work should strive to elucidate how specific associations between and within perceptual modalities are driven differentially by statistical, structural, and linguistic factors.

## Supplemental Material

sj-docx-1-ipe-10.1177_20416695211048513 - Supplemental material for Crossmodal Associations with Olfactory, Auditory, and Tactile Stimuli in Children and Adults Click here for additional data file. Supplemental material, sj-docx-1-ipe-10.1177_20416695211048513 for Crossmodal Associations with Olfactory, Auditory, and Tactile Stimuli in Children and Adults by Laura J. Speed, Ilja Croijmans, Sarah Dolscheid and Asifa Majid in i-Perception

## Notes

1. Note that crossmodal associations are distinct from canonical synaesthesia (Deroy & Spence, 2013b). Synaesthesia is a rare neurological phenomenon where individuals experience automatic, vivid, and idiosyncratic associations between the senses. In contrast, crossmodal associations are frequent in the population, are not typically associated with conscious perception, and are said to be regular and predictable (Deroy & Spence, 2013b).

## Appendix A

**Table A1.** Experiment 1 Pitch Associations.

			Angularity	Brightness	Height	Sharpness	Size	Thickness	Weight
4–5 years	High	*z*	0.71	0.45	1	2.45	1	1	0
		*p*	.48	.66	.32	.01	.32	.32	1
	Low	*z*	0.71	0.45	1.13	1.34	0.33	2	0.63
		*p*	.48	.66	.26	.18	.74	.05	.53
6–9 years	High	*z*	2.08	5	0.67	2.29	3.02	2.47	2.21
		*p*	.04	<.001*	.51	.022	.002*	.014	.27
	Low	*z*	3.8	0.62	0.32	1.51	1.33	1.18	2.11
		*p*	<.001*	.54	.75	.13	.18	.24	.035
10–18 years	High	*z*	2.78	6.35	1.95	4.52	6.12	7.3	4.37
		*p*	.005*	<.001*	.051	<.001*	<.001*	<.001*	<.001*
	Low	*z*	4.39	1.61	1.36	4.62	4.36	5.9	3.71
		*p*	<.001*	.11	.17	<.001*	<.001*	<.001*	<.001*
19+ years	High	*z*	5.03	8.09	8.03	6.87	8.43	8.72	7.12
		*p*	<.001*	<.001*	<.001*	<.001*	<.001*	<.001*	<.001*
	Low	*z*	9.26	3.18	1.48	6.74	4.25	6.04	4.25
		*p*	<.001*	.001*	.14	<.001*	<.001*	<.001*	<.001*

*Note*. Asterisks indicate associations greater than chance according to Wilcoxon Signed Rank Tests.

**Table A2.** Experiment 1 Pitch Associations. Effect of the Pitch for Each Dimension and Age Group.

		Thickness	Size	Height	Brightness	Angularity	Sharpness	Weight
4−5 years	*z*	1.90	0.81	1.41	0.0	0.0	2.50	0.82
	*p*	.06	.01	.16	1	1	.01	.42
6–9 years	*z*	2.28	2.74	0.64	3.20	3.81	2.49	2.96
	*p*	.02	.006*	.52	.001*	<.001*	.01	.003*
10–18 years	*z*	7.33	6.22	0.39	5.17	4.38	5.20	4.76
	*p*	<.001*	<.001*	.70	<.001*	<.001*	< .001*	<.001*
19+ years	*z*	8.74	7.70	6.17	7.37	9.62	8.48	7.42
	*p*	<.001*	<.001*	<.001*	<.001*	<.001*	<.001*	<.001*

*Note*. Asterisks indicate significance at the correct alpha level of .0125

## Appendix C. Statistics Experiment 3

**Table B1.** Experiment 2. Associations Between Haptic Texture (Hard–Soft, Rough–Smooth, Pointed–Rounded) and Pitch and Shape.

			Pitch	Shape				Pitch	Shape
4–5 years	Hard	*t*	0.33	1.80	4–5 years	Rough	*t*	−1.23	0.89
		*p*	.75	.11			*p*	.26	.40
	Soft	*t*	0.81	0.94		Smooth	*t*	−1.34	0.97
		*p*	.44	.38			*p*	.22	.36
6–9 years	Hard	*t*	0.25	5.09	6–9 years	Rough	*t*	0.16	−4.64
		*p*	.80	<.001*			*p*	.87	<.001*
	Soft	*t*	0.33	6.78		Smooth	*t*	−2.36	0.39
		*p*	.74	<.001*			*p*	0.02	.70
10–18 years	Hard	*t*	1.84	4.12	10–18 years	Rough	*t*	2.98	−10.98
		*p*	.07	<.001*			*p*	.004*	<.001*
	Soft	*t*	1.46	11.13		Smooth	*t*	−2.75	1.27
		*p*	.15	<.001*			*p*	.007*	.21
19+ years	Hard	*T*	4.60	8.08	19+ years	Rough	*t*	1.66	−16.80
		*p*	<.001*	<.001*			*p*	.10	<.001*
	Soft	*t*	4.16	19.40		Smooth	*t*	−5.58	.58
		*p*	<.001*	<.001*			*p*	< .001*	.57
4–5 years	Pointed	*t*	0.34	1.87					
		*p*	.75	.10					
	Rounded	*t*	−0.37	3.22					
		*p*	.72	.01					
6–9 years	Pointed	*t*	−1.89	−2.34					
		*p*	.06	.02					
	Rounded	*t*	−0.55	5.36					
		*p*	.59	<.001*					
10–18 years	Pointed	*t*	3.09	−3.28					
		*p*	.002*	.001*					
	Rounded	*t*	−0.86	9.84					
		*p*	.39	<.001*					
19+ years	Pointed	*t*	−5.40	−3.55					
		*p*	<.001*	<.001*					
	Rounded	*t*	1.71	15.60					
		*p*	.09	<.001*					

*Note*. Asterisks indicate associations significantly different to chance.

**Table B2.** Significant Hue Associations to Haptic Texture With Proportion of Choices in Each Age Group.

	4–5 years						6–9 years			
	Color	Prop.	*z*	*p*			Color	Prop.	*z*	*p*
Hard						Hard	Grey	0.2	5.67	<.001
Soft						Soft	Grey	0.2	4.46	<.001
Rough						Rough	Grey	0.3	8.94	<.001
							Yellow	0.2	3.31	<.001
Smooth						Smooth	Grey	0.2	4.06	<.001
							White	0.3	16.77	<.001
Pointed						Pointed	White	0.4	25.66	<.001
Rounded	White	0.3	5.52	<.001		Rounded	White	0.6	41.95	<.001
	10–18 years				* *		19+ years			
Hard	Grey	0.2	6.85	<.001		Hard	Grey	0.2	7.74	<.001
Soft	White	0.1	4.16	<.001		Soft	Red	0.2	5.26	<.001
Rough	Grey	0.2	4.27	<.001		Rough	Grey	0.2	5.91	<.001
	Yellow	0.2	4.05	<.001			Yellow	0.2	4.92	<.001
	Yellow–red	0.3	8.00	<.001			Yellow–red	0.4	13.75	<.001
Smooth	Purple–blue	0.3	6.68	<.001		Smooth	Purple–blue	0.2	6.55	<.001
	Grey	0.2	4.64	<.001			Grey	0.2	6.43	<.001
	White	0.2	12.93	<.001			White	0.2	15.67	<.001
Pointed	White	0.4	29.73	<.001		Pointed	White	0.3	24.22	<.001
Rounded	White	0.5	34.43	<.001		Rounded	White	0.4	36.10	< .001

*Note*. Empty cells indicate no colors had significant associations.

## Appendix

**Table C1.** Associations Between Odor and Pitch, Odor and Shape, and Odor and Texture.

			Pitch	Shape	Texture
3–5 years	Caramel	*t*	1.03	.95	.69
		*p*	.32	.36	.5
		*d*	.29	.26	.19
	Menthol	*t*	2.91	1.95	.42
		*p*	.01*	.08	.69
		*d*	.81	.54	.12
	Raspberry	*t*	1.27	.24	.68
		*p*	.22	.81	.51
		*d*	.36	.07	.19
	Lemon	*t*	.12	.62	.94
		*p*	.9	.55	.37
		*d*	.03	.17	.26
	Onion	*t*	.13	1.24	.35
		*p*	.09	.24	.73
		*d*	.04	.34	.1
6–9 years	Caramel	*t*	1.2	4.81	5.68
		*p*	.23	<.001*	<.001*
		*d*	.13	.12	.60
	Menthol	*t*	1.54	2.92	0.52
		*p*	.13	.004*	.60
		*d*	.16	.31	.06
	Raspberry	*t*	2.45	1.16	4.13
		*p*	.02	.25	<.001*
		*d*	.26	.12	.44
	Lemon	*t*	1.79	1.53	.47
		*p*	.08	.13	.64
		*d*	.19	.16	.05
	Onion	*t*	1.72	.79	2.85
		*p*	.09	.43	.005*
		*d*	.18	.08	.3
10–18 years	Caramel	*t*	1.11	7	9.07
		*p*	.27	<.001*	<.001*
		*d*	.10	.75	.85
	Menthol	*t*	2.13	1.28	3.16
		*p*	.04	.20	.002*
		*d*	.2	.12	.3
	Raspberry	*t*	3.62	6.87	6.26
		*p*	<.001*	<.001*	<.001*
		*d*	.34	.65	.59
	Lemon	*t*	6.81	.59	2.84
		*p*	<.001*	.56	.005*
		*d*	.64	.06	.27
	Onion	*t*	3.43	.55	5.98
		*p*	.001*	.59	<.001*
		*d*	.32	.05	.56
19+ years	Caramel	*t*	.98	14.41	10.91
		*p*	.33	<.001*	<.001*
		*d*	.06	.93	.71
	Menthol	*t*	5.3	3.17	10.91
		*p*	<.001*	.002*	<.001*
		*d*	.34	.21	.2
	Raspberry	*t*	1.09	4.55	10.93
		*p*	.28	<.001*	<.001*
		*d*	.07	.29	.71
	Lemon	*t*	9.36	1.98	4.01
		*p*	<.001*	.05	<.001*
		*d*	.61	.13	.26
	Onion	*t*	4.93	1.16	7.69
		*p*	<.001*	.25	<.001*
		*d*	.32	.07	.5

*Note*. Asterisks indicate associations significantly different to chance.

**Table C2.** Significant Odor–Hue Associations.

	4–5 years					6–9 years			
	Color	Prop	*z*	*p*		Color	Prop	*z*	*p*
Caramel	Yellow–red	0.38	3.81	<.001	Caramel	Red–purple	0.21	3.28	.001
						Yellow–red	0.21	5.02	<.001
Menthol	White	0.08	5.56	<.001	Menthol	White	0.04	14.9	<.001
Raspberry					Raspberry	Red	0.16	2.93	.002
Lemon	Yellow	0.46	4.72	<.001	Lemon	Yellow	0.12	8.17	<.001
Onion					Onion	Yellow	0.2	3.28	<.001
						Yellow–red	0.38	8.87	<.001
	10–18 years				* *	19 + years			
Caramel	Yellow-red	0.21	10.6	<.001	Caramel	Yellow	0.15	3.5	0.009
	White	0.09	4	<.001		Yellow–red	0.3	20.88	<.001
Menthol	White	0.05	13	<.001	Menthol	Green	0.08	9.86	<.001
						White	0.06	6.36	<.001
Raspberry	Red	0.26	6.61	<.001	Raspberry	Red	0.24	9.72	<.001
	Red–purple	0.17	3.17	.001					
Lemon	Yellow	0.08	11.93	<.001	Lemon	Yellow	0.13	19.59	<.001
						Green–yellow	0.09	2.64	<.001
Onion	Yellow	0.2	3.79	<.001	Onion	Yellow–red	0.36	16.8	<.001
	Yellow–red	0.35	9.42	<.001					
