# Supplementary material for: Crossmodal Associations with Olfactory, Auditory, and Tactile Stimuli in Children and Adults
Source: Iperception. 2021 Dec 6;12(6):20416695211048513. doi: 10.1177/20416695211048513 (PMC8652194; doi:10.1177/20416695211048513)
Supplement: sj-docx-1-ipe-10.1177_20416695211048513 - Supplemental material for Crossmodal Associations with Olfactory, Auditory, and Tactile Stimuli in Children and Adults [file sj-docx-1-ipe-10.1177_20416695211048513.docx]

**Supplementary Data**

**Experiment 1: Pitch**

To assess whether choices differed across age, ordinal regression was conducted since the dependent variable is ordinal (counts 0, 1, or 2) and the predictor variable is continuous (age). We conducted ordinal regressions on counts for high and low pitch separately on each dimension. Again, a Bonferroni-adjusted *p*-value of .004 was applied. Mappings between high pitch and high space, increased with age (see Table 2); but age was not a predictor for any of the other mappings.

Table 2. Ordinal regression with predictor age on each dimension. Asterisks denote p < .004 (Bonferroni-corrected alpha).

|  | High | | | | Low | | | |
| --- | --- | --- | --- | --- | --- | --- | --- | --- |
|  | *B* | *SE* | *Wald* | *p* | *B* | *SE* | *Wald* | *p* |
| Thickness | .001 | .005 | .020 | .89 | .002 | .005 | .089 | .77 |
| Size | .006 | .005 | 1.42 | .23 | .004 | .005 | .547 | .46 |
| Height | .016 | .005 | 9.85 | .002* | -.005 | .005 | .952 | .33 |
| Brightness | -.003 | .005 | .342 | .56 | -.007 | .005 | 1.69 | .19 |
| Angularity | -.001 | .005 | .063 | .80 | -.044 | .005 | 4.50 | .03 |
| Sharpness | -.004 | .005 | .480 | .49 | -.006 | .005 | 1.29 | .26 |
| Weight | .007 | .005 | 1.78 | .18 | .007 | .005 | 1.84 | .18 |

**Experiment 2: Texture**

To assess whether texture-pitch ratings differed with age, separate linear regressions were conducted for each texture separately, with age as predictor. With an adjusted alpha level of 0.008, pitch ratings did not differ according to age for any textures (see Table 3).

Table 3. Linear regression with predictor age on pitch ratings for each texture.

|  | *B* | *SE* | *β* | *t* | *p* |
| --- | --- | --- | --- | --- | --- |
| hard | .12 | .085 | .064 | 1.34 | .18 |
| soft | .15 | .089 | .079 | 1.66 | .10 |
| rough | .034 | .092 | .018 | .37 | .71 |
| smooth | .010 | .085 | 006 | .12 | .91 |
| sharp | -.093 | .086 | -.052 | -1.09 | .28 |
| blunt | .179 | .087 | .098 | -2.06 | .04 |

Using the adjusted alpha level of 0.008, shape ratings did not differ by age for any textures (see Table 4).

Table 4. Linear regression with predictor age on shape ratings for each texture.

|  | *B* | *SE* | *β* | *t* | *p* |
| --- | --- | --- | --- | --- | --- |
| hard | -.030 | .082 | -.017 | -.37 | .72 |
| soft | .101 | .073 | .066 | 1.38 | .17 |
| rough | -.171 | .077 | -.106 | -2.23 | .03 |
| smooth | -.032 | .093 | -.017 | -.35 | .73 |
| sharp | .024 | .087 | .013 | .28 | .78 |
| blunt | .029 | .074 | .019 | .40 | .69 |

**Hue**

If a color was significantly matched to a texture, loglinear regression was conducted to test whether choices differed by age. An adjusted alpha was used based on the number of tests used per texture. The only mappings predict by age were grey for the soft texture and the white color matches for the sharp and blunt textures.

|  |  | Color | *B* | *SE* | *Wald* | *p* |
| --- | --- | --- | --- | --- | --- | --- |
|  | Hard | yellow  grey | -.013  -.001 | .009  .006 | 2.18  .922 | .14  .27 |
|  | Soft | grey  red  white | -.033  .012  .003 | .010  .007  .010 | 10.09  3.32  .098 | .001*  .07  .75 |
|  | Rough | grey | -.003 | .007 | .259 | .61 |
|  |  | yellow | -.003 | .007 | .187 | .67 |
|  |  | yellow-red | .013 | .006 | 5.03 | .03 |
|  | Smooth | purple-blue | .002 | .006 | .151 | .70 |
|  |  | grey | -.002 | .007 | .068 | .79 |
|  |  | white | -.008 | .007 | 1.31 | .25 |
|  | Sharp | white | -.022 | .006 | 12.15 | < .001* |
|  | Blunt | white | -.026 | .006 | 20.06 | < .001* |
|  |  |  |  |  |  |  |

**Chroma**

To test an effect of age on chroma, linear regressions were conducted since both age and chroma are continuous variables. Separate linear regressions with age as predictor were conducted for each texture separately, with a Bonferroni-adjusted alpha value of 0.008 (i.e., 0.05/6 regressions). Colors chosen for the soft, sharp, and blunt texture increased in chroma with age.

Table 5. Linear regression with predictor age on chroma for each texture. Asterisks denote Bonferroni-adjusted alpha level of 0.008.

|  | *B* | *SE* | *β* | *t* | *p* |
| --- | --- | --- | --- | --- | --- |
| hard | .005 | .065 | .004 | .08 | .94 |
| soft | .223 | .063 | .166 | 3.52 | <.001* |
| rough | .034 | .067 | .024 | .451 | .61 |
| smooth | .098 | .063 | .074 | 1.55 | .12 |
| sharp | .225 | .074 | .144 | 3.05 | .002* |
| blunt | .439 | .068 | .293 | 6.42 | <.001* |

**Lightness**

Separate linear regressions with age as predictor were conducted for each texture separately with a Bonferroni-adjusted alpha value of 0.008 (i.e., 0.05/6 regressions). Colors chosen for the sharp and blunt texture decreased in lightness with age.

Table 6. Linear regression with predictor age on lightness for each texture. Asterisks denote Bonferroni-adjusted alpha level of 0.008.

|  | *B* | *SE* | *β* | *t* | *p* |
| --- | --- | --- | --- | --- | --- |
| hard | .048 | .065 | .035 | .73 | .47 |
| soft | .054 | .063 | .041 | .86 | .38 |
| rough | -.082 | .054 | -.073 | -1.54 | .12 |
| smooth | -.007 | .061 | -.005 | -.11 | .91 |
| sharp | -.236 | .060 | -.185 | -3.94 | < .001* |
| blunt | -.204 | .054 | -.178 | -3.79 | < .001* |

**Experiment 3: Odor**

**Pitch**

Separate linear regressions were conducted for each odor with age as a predictor. Pitch ratings did not differ for any odor across age.

Table 7. Linear regression with predictor age on pitch ratings for each odor.

|  | *B* | *SE* | *β* | *t* | *p* |
| --- | --- | --- | --- | --- | --- |
| caramel | -.114 | .083 | -.064 | -1.36 | .17 |
| lemon | .143 | .080 | .084 | 1.79 | .07 |
| menthol | .058 | .077 | .036 | .75 | .45 |
| onion | -.038 | .087 | -.020 | -.43 | .67 |
| raspberry | -.093 | .082 | -.053 | -1.13 | .26 |

**Shape**

Separate linear regressions were conducted for each odor with age as a predictor, with the adjusted *p*-value of .01. There were no effects of age on shape ratings for any odor.

Table 8. Linear regression with predictor age on shape ratings for each odor. Asterisks denote Bonferroni-adjusted p < .01.

|  | *B* | *SE* | *β* | *t* | *p* |
| --- | --- | --- | --- | --- | --- |
| caramel | .154 | .068 | .106 | 2.25 | .03 |
| lemon | -.204 | .084 | -.114 | -2.43 | .02 |
| menthol | -.113 | .081 | -.065 | -1.39 | .17 |
| onion | -.052 | .092 | -.027 | -.57 | .57 |
| raspberry | -.117 | .079 | -.070 | -1.48 | .14 |

**Texture**

Separate linear regressions were conducted for each odor with age as a predictor, but age did not significantly predict texture ratings for any odor.

Table 9. Linear regression with predictor age on shape ratings for each odor.

|  | *B* | *SE* | *β* | *t* | *p* |
| --- | --- | --- | --- | --- | --- |
| caramel | -.012 | .076 | -.007 | -.16 | .88 |
| lemon | -.023 | .083 | -.013 | -.27 | .79 |
| menthol | .000 | .084 | .000 | -.003 | 1.0 |
| onion | .024 | .083 | .013 | .28 | .78 |
| raspberry | .005 | .077 | .003 | .07 | .95 |

**Hue**

If a color was significantly matched to a odor, logistic regression was conducted to test whether choices differed by age. An adjusted alpha was used based on the number of tests used per odor. No mappings were predicted by age.

|  |  | Color | *B* | *SE* | *Wald* | *p* |
| --- | --- | --- | --- | --- | --- | --- |
|  | Caramel | red-purple  yellow  yellow-red  white | -.008  -.007  .003  -.007 | .008  .008  .005  .012 | .987  .914  .406  .382 | .32  .34  .53  .54 |
|  | Lemon | green-yellow  yellow | .009  .010 | .007  .005 | 1.40  3.69 | .24  .06 |
|  | Onion | grey | .001 | .008 | .017 | .90 |
|  |  | yellow | -.001 | .007 | .028 | .87 |
|  |  | yellow-red | .006 | .005 | 1.33 | .25 |
|  | Menthol | green  yellow-red | -.009  -.004 | .007  .008 | 1.63  .228 | .20  .63 |
|  |  | white | -.011 | .007 | 2.33 | .13 |
|  | Raspberry | red  red-purple | .001  -.001 | .006  .008 | .014  .015 | .91  .90 |
